# Lamin A and the LINC complex act as potential tumor suppressors in Ewing Sarcoma

**DOI:** 10.1038/s41419-022-04729-5

**Published:** 2022-04-14

**Authors:** Francesca Chiarini, Francesca Paganelli, Tommaso Balestra, Cristina Capanni, Antonietta Fazio, Maria Cristina Manara, Lorena Landuzzi, Stefania Petrini, Camilla Evangelisti, Pier-Luigi Lollini, Alberto M. Martelli, Giovanna Lattanzi, Katia Scotlandi

**Affiliations:** 1 CNR Institute of Molecular Genetics “Luigi Luca Cavalli-Sforza”, Unit of Bologna, 40136 Bologna, Italy; 2 IRCCS Istituto Ortopedico Rizzoli, 40136 Bologna, Italy; 3 Alma Mater Studiorum, University of Bologna, Department of Biomedical and Neuromotor Sciences, 40136 Bologna, Italy; 4 IRCCS Istituto Ortopedico Rizzoli, Experimental Oncology Laboratory, 40136 Bologna, Italy; 5 Alma Mater Studiorum, University of Bologna, Department of Experimental, Diagnostic and Specialty Medicine, 40138 Bologna, Italy; 6 Confocal Microscopy Core Facility, Research Center, Bambino Gesu’ Children’s Hospital IRCCS, 00146 Rome, Italy

**Keywords:** Nuclear organization, Cancer

## Abstract

Lamin A, a main constituent of the nuclear lamina, is involved in mechanosignaling and cell migration through dynamic interactions with the LINC complex, formed by the nuclear envelope proteins SUN1, SUN2 and the nesprins. Here, we investigated lamin A role in Ewing Sarcoma (EWS), an aggressive bone tumor affecting children and young adults. In patients affected by EWS, we found a significant inverse correlation between *LMNA* gene expression and tumor aggressiveness. Accordingly, in experimental in vitro models, low lamin A expression correlated with enhanced cell migration and invasiveness and, in vivo, with an increased metastatic load. At the molecular level, this condition was linked to altered expression and anchorage of nuclear envelope proteins and increased nuclear retention of YAP/TAZ, a mechanosignaling effector. Conversely, overexpression of lamin A rescued LINC complex organization, thus reducing YAP/TAZ nuclear recruitment and preventing cell invasiveness. These effects were also obtained through modulation of lamin A maturation by a statin-based pharmacological treatment that further elicited a more differentiated phenotype in EWS cells. These results demonstrate that drugs inducing nuclear envelope remodeling could be exploited to improve therapeutic strategies for EWS.

## Introduction

Ewing Sarcoma (EWS) is the second most common type of primary bone tumor and mainly affects children and adolescents. It is a very aggressive and poorly differentiated neoplasia, with high tendency to lung and/or bone metastatization [1, 2]. While progresses in multimodal therapy of patients with localized disease have significantly increased survival up to 65-70%, the prognosis for patients with metastatic or recurrent EWS remains very poor, independently of intensification of chemotherapeutic regimens. Moreover, a significant proportion of EWS patients suffer from toxic effects of chemotherapy, or eventually die, due to tumor progression or relapse [2]. Therefore, a deeper understanding of the biology underlying metastatic process, and the identification of reliable risk- and response-related biomarkers of patient survival are urgent needs for the development of novel and more effective therapeutic options.

Aside from molecular alterations, nuclear shape and size and chromatin conformation can provide parameters for cancer diagnostics, but the functional significance of these alterations in the context of EWS disease progression and metastatic processes is not yet fully clarified [3, 4].

Nuclear envelope ruptures occur during cancer cell migration and enable the passage of cancer cells through small gaps [5]. These observations support the concept that shape instability of the nuclear envelope is functionally relevant in aggressive malignancies, and has potential consequences for genome instability, tumor progression, and metastatization [5–8]. Nuclear envelope proteins play a key role in a variety of cellular pathways implicated in tumorigenesis and their altered expression has been detected in human cancers, often associated to an aggressive phenotype [4, 9, 10]. The nuclear envelope is formed by the nuclear membrane and the nuclear lamina, the latter being located beneath the inner nuclear membrane. A type lamins, including lamin A and C, splicing products of the *LMNA* gene, are main constituents of the nuclear lamina [11, 12]. Lamin A is translated as a precursor protein called prelamin A, which undergoes rapid post-translational processing and maturation, a process that can be partially inhibited by statin treatment [13]. Lamin A maintains nuclear architecture, constitutes platforms for chromatin and transcription factor tethering, and is involved in the connection between the nucleus and the cytoskeleton, which is essential for mechanosignaling [14, 15]. Nuclear mechanosignaling is carried out through the LINC complex, a dynamic platform formed by nuclear envelope proteins as SUN1, SUN2, and the nesprins that connect the nucleoskeleton to the cytoskeleton [16, 17]. It has been demonstrated that expression of *LMNA* gene scales with tissue stiffness, while cancer cell sensitivity to mechanical stress is related to *LMNA* levels [8, 18].

Lamin alterations may decrease nuclear rigidity, thereby promoting invasiveness, or increase protection against mechanical forces, such as increased interstitial pressure within the tumor, or alter gene expression [19, 20].

It has been observed that the expression of lamin A can be increased, reduced or absent, depending on the type of cancer, its aggressiveness, proliferation, and degree of differentiation [21–23]. Decrease in lamin A levels is often associated with poor prognosis in multiple human cancers [11]. However, elevated lamin A levels have been also observed in some tumors [24] [25].

In this study, we report that *LMNA* gene is significantly downregulated in metastatic lesions of EWS patients as compared to primary tumors, while low *LMNA* transcription is directly correlated with poor survival. Our data show that low lamin A levels are linked to increased EWS cell migration and invasiveness caused by an impaired mechanosignaling. Mechanistically, in EWS cells, reduced lamin A levels impair anchorage of LINC complex proteins and support an invasive cellular phenotype, while induction of lamin A or prelamin A, either by molecular or pharmacological approaches, rescues LINC complex proteins and cytoskeleton-related cellular signaling effectors YAP/TAZ, while inhibiting cell migration and invasiveness. After intravenous injection in immunodeficient mice, cells overexpressing lamin A gave rise to a significantly lower metastatic load to the liver. As a whole, our findings demonstrate that modulation of lamin A levels can be exploited to decrease invasiveness of EWS cells, paving the way to new therapeutic options for this very aggressive cancer.

## Materials and Methods

### Preclinical studies

#### Cell lines and culturing

Human EWS cell lines authentication was executed by short tandem repeat (STR) polymerase chain reaction (PCR) analysis using a PowerPlex ESX Fast System kit (Promega, Madison, WI, USA) and the last control was performed in December 2017. All cell lines were tested for mycoplasma contamination every three months using MycoAlert mycoplasma detection kit (Lonza, Basel, Switzerland). IOR/CAR and LAP-35 cells were immortalized in the Experimental Oncology Laboratory, Rizzoli Orthopaedic Institute of Bologna [26]. Cultures were grown in Iscove’ Modified Dulbecco’s Medium (IMDM, Thermo Fisher, Milan, Italy) supplemented with 10% of heat-inactivated fetal bovine serum (FBS, Life Technologies, Monza, Italy), 2mM l-glutamine, 100 U/mL penicillin and 100 μg/mL streptomycin (Sigma-Aldrich, Saint Louis, MO, USA) at 37 °C in a humidified atmosphere of 5% CO_2_.

### Differentiation of TC-71 cell line

TC-71 cells were seeded at low density in IMDM with FBS 1% for 72 hours and then evaluated for neural markers as reported elsewhere [27]. At least three independent experiments were performed.

### *LMNA* silencing/overexpression

*LMNA* silencing was done by employing siRNAs duplexes specific for human *LMNA* (siRNA *LMNA*) purchased from Thermo Fisher. Scrambled duplexes were used as control (silencer select negative control: 4390842 and 439043). EWS cells were plated in 6-well plates (0.3 × 10^6^ cells per well) and, at approximately 50% confluence, cells were transfected with the annealed siRNA-*LMNA* and siRNA-scrambled using lipofectamine 3000 (Thermo Fisher). The experiments were performed after 24, 48, 72, and 96 hours from transfection.

TC-71 cells were stably transfected with Empty-GFP and Lamin A-GFP plasmids (Clontech, Mountain View, CA) carrying the Enhanced Green Fluorescent (EGFP) gene and a neomycin resistance gene expression cassette. In this study, we employed the following stably transfected clones: Empty-GFP vector #2, lamin A-GFP #30-40 and lamin A-GFP #84.

Overexpression of unprocessable prelamin A (LA-C661M) was obtained as reported elsewhere [28].

### Western blotting

Western blotting was performed by standard methods. Briefly, cell pellets were lysed in RIPA buffer (containing 20 mM Tris-HCl (pH 7.5), 150 mM NaCl, 1 mM EDTA, 1 mM EGTA, 1% NP-40, 0.5% sodium deoxycholate, 2.5 mM sodium pyrophosphate, 1 mM β-glycerophosphate, 1 mM, Na3VO4, 0.1% sodium dodecyl sulfate) and protease inhibitors (from Thermo Fisher). After sonication, the total lysate was resolved by SDS-PAGE, employing Criterion TGX polyacrylamide gels (Bio-Rad, Hercules, CA, USA). Proteins were then blotted onto nitrocellulose membranes (Bio-Rad) and immunoblotted with the antibodies indicated in Table 1. Proteins were detected using the Cyanagen Westar ECL western blotting detection reagent (Cyanagen Bologna, Italy), the ChemiDoc-It2 Imaging System, and the Vision Works LS Software for the analysis (UVP, LLC, Upland, CA, USA). Uncropped Western blot images are available in the supplemental material.

**Table 1:** Antibodies used for IF and Western Blotting.

Antibody	Product code	Company	Application	Species
LAMIN A/C	#4777	CST	WB, ICC	Mouse
GAPDH	#5174	CST	WB	Rabbit
ROCK2	PA5-78290	Invitrogen	WB	Rabbit
β-ACTIN	#4970	CST	WB	Rabbit
SUN1	HPA008346	Sigma-Aldrich	WB, ICC	Rabbit
SUN2	HPA001209	Sigma-Aldrich	WB, ICC	Rabbit
EMERIN	#30853	CST	WB, ICC	Rabbit
NESPRIN2 G	#IQ562	IMMUQUEST	ICC	Mouse
PRELAMIN A	MABT858	Merck Millipore	WB	Mouse
YAP	#14074	CST	WB, ICC	Rabbit
Phospho-YAP	#13008	CST	WB	Rabbit
MYC	#18583	CST	WB	Rabbit
β3-TUBULIN	T5076	Sigma-Aldrich	ICC	Mouse
NEUROFILAMENT-H	#2836	CST	WB, ICC	Mouse

### Immunofluorescence/Confocal microscopy

To assess the expression and subcellular localization of the proteins of interest, EWS cells were seeded in 12-well plates and the cells were grown on coverslips, fixed with methanol (100%) for 8 min. TC-71 cells were seeded on fibronectin-coated coverslips and first fixed in paraformaldehyde 4% in PBS, then permeabilized with methanol (100%, 8 min).

Cells were then blocked with 3% BSA-containing PBS for 1 h. Antibodies diluted in 3% BSA-containing PBS were applied overnight at 4 °C and revealed by using secondary antibodies diluted 1:300 (incubated for 1 hour at RT) from Thermo Fisher. The antibodies used are listed in Table 1. Samples were mounted with a DAPI-containing anti-fade reagent (Molecular Probes, Thermo Fisher) and observed with a Nikon Eclipse Ni epifluorescence microscope. The images captured with NIS-Elements 4.3 AR software were processed using Photoshop CS6 (Adobe Systems, Inc., San Jose, CA, USA).

Confocal microscopy was performed on a Leica TCS-SP8X laser-scanning confocal microscope (Leica Microsystems, Mannheim, Germany) equipped with tunable white light laser (WLL) source, 405 nm diode laser, 3 Internal Spectral Detector Channels (PMT), and 2 Internal Spectral Detector Channels (HyD) GaAsP. Sequential confocal images were acquired using an HCPLAPO 63x oil-immersion objective (1.40 numerical aperture, NA, Leica Microsystems). Acquisition settings (i.e. lasers’ power, beam splitters, filter settings, pinhole diameters and scan mode) were the same for all examined samples of each staining. Tables of images were processed using Adobe Photoshop CS6 software (Adobe Systems Inc).

### Migration and invasion assays

Cell migration and invasion assay kits were purchased by Cell Biolabs (CytoSelect assays) and were employed to analyze the migratory and invasive properties of EWS cells. For migration assays, 8 μm pore size polycarbonate membrane inserts were used, while for invasion studies, polycarbonate membrane inserts with the upper surface coated with a uniform layer of basement membrane matrix solution were employed. Assays were performed according to manufacturer’s protocol. Briefly, 500 µL of IMDM containing 20% FBS were added to the lower chambers. Cells were resuspended in IMDM with 1% FBS and seeded on the top of the membrane of each well. A-673 cells were seeded at final density of 200,000 or 100,000 cells for invasion and migration assays, respectively. Parental TC-71 and TC-71 clones overexpressing lamin A were seeded at a final density of 200,000 cells for both migration and invasion assays. LAP-35 were seeded at final density of 100,000 cells for migration assay. For each experimental point we performed at least three replicates. Cells were incubated for 24 and 48 h, at 37 °C in 5% CO_2_ in a humidified atmosphere.

Pictures were taken at different time points with Olympus CKX41 microscope to assess the presence of migrated cells. Then, Extraction Solution was added and incubated for 15 min, to allow cell lysis and the OD at 570 nm was measured in an ELISA plate reader (Bio-Rad). Data were plotted as the percentage of silenced/or overexpressed migrated cells versus scramble or empty GFP migrated cells.

### Wound healing assay

EWS cells were seeded in 24-well plates and wound healing assays were performed. A reproducible longitudinal scratch was made in a confluent monolayer, and the wound closure was assessed at 0 and 24 h by photographing the central field of the scratches under an inverted light microscopy (Olympus CKX41, Olympus Corp, Tokyo, Japan) mounted with a digital camera (C-7070 Wide Zoom, Olympus) at 10X magnification. Morphometric analysis of cell migration was performed using a computerized image analysis system (Qwin, 3.0 software, Leica Microsystem Imaging Solution, Ltd., Wetzlar, Germany). A region that included the artificial scratch and the adjacent cell monolayer was selected as the standard region of interest (ROI). The wound closure was calculated as (1-Ax/A0) %, where A0 and Ax represented the empty scratch area at 0 and 24 h, respectively.

### Real-time (RT)-PCR analysis

Total RNA was extracted using the RNeasy Mini Kit (Qiagen, Venlo, The Netherlands) according to the manufacturer’s instructions and 500 ng of total RNA was reverse transcribed using High-Capacity cDNA Reverse Transcription Kit (Thermo Fisher). Gene expression was assessed using the TaqMan® Gene Expression Master Mix, and predesigned TaqMan probes (Thermo Fisher) *LMNA* (Hs.PT.58.24496716), nestin gene (Hs.PT.58.1185097), *SOX2* (Hs.PT.58.237897.g), Connective Tissue Growth Factor (*CTGF*) (Hs00170014), Cysteine Rich Angiogenic Inducer 61 *(CYR61*) (Hs00155479), Neurofilament H (*NEF-H*) (Hs00606024) and *β3-tubulin*gene (Hs.PT.58.20385221) were employed, using the 7300 real-time PCR system (Applied Biosystems, Foster City, CA, USA).

Relative quantification was performed using the ΔCT method (relative abundance, RA = 2^- ΔCT^) or the ΔΔCT method (relative quantification, RQ = 2^- ΔΔCT^). The expression levels of the target genes were normalized to those of the housekeeping gene *GAPDH* (Hs99999905_m1).

### Chemicals

5-Azacytidine and Mevinolin were purchased from Sigma-Aldrich, Saint Louis, MO, USA.

5-Azacytidine was employed at 4 μM for 24 hours in TC-71, IOR/CAR and A-673 cell lines. Mevinolin was used at 2.5, 5, 7.5, 10 μM for 24, 48 hours.

### Patients

We firstly analyzed original microarray data of EWS samples, available at gene expression omnibus (GEO) with accession number GSE17679 (probe 203411_s_at) [29]. This cohort consists of 64 Ewing patients for which gene expression profiles in EWS tumors were analyzed with HG-U133 plus 2.0 microarrays (Affymetrix, Santa Clara, CA, USA). The gene expression values for *LMNA* gene were plotted for metastatic samples and primary samples, evaluating significant differences between the two groups. We also employed microarray data of 64 EWS patients with clinical data available at GSE63157 (Human Exon 1.0 ST Array) and we created a 5 years overall survival curve (Kaplan-Meier) according to the median value of *LMNA* gene [30].

### Immunohistochemistry

Avidin–biotin–peroxidase method was used for lamin A immunostaining in four representative EWS samples from GSE17679 [29] (patients R72, R80 R48, R29) to evaluate nuclear shape in relation to *LMNA* expression. Antigen retrieval was performed using citrate buffer (pH 6.0), prior to incubation with the anti-lamin A/C (E-1, mouse, sc-376248 dilution 1:100; Santa Cruz Biotechnology, Dallas, TX, USA). Images were taken with a Nikon Microscope, at 100X magnification.

To measure nuclear circularity, the contour ratio algorithm was used and calculated by following the formula: Contour ratio=4π x nuclear area/nuclear perimeter^2^. NIS-Elements 4.3 AR software was employed to calculate nuclear circularity. For each EWS sample analyzed, at least 100 nuclei were measured.

### In vivo studies

Immunodeficient double knockout BALB/c Rag2-/-;Il2rg-/- breeders were kindly provided by the Central Institute for Experimental Animals (Kawasaki, Japan) [31].

Mice were bred in the Animal Care Facility of the Laboratory of Immunology and Biology of Metastasis, (University of Bologna). TC-71 parental cells and the stably transfected clones, Empty-GFP vector #2, lamin-GFP #30-40 and lamin-GFP #84 were injected intravenously (iv) (2× 10^6^ cells/mouse, *n* = 7 male mice, 19-33-week-old, for each group) to assess experimental metastasis as previously reported [32]. The human TC-71 and its stably transfected clones injected iv in immunodeficient mice represent a model of cell line-derived xenograft (CDX) for the study of experimental metastasis, at the moment, to the best of our knowledge, EWS models of mouse origin that could better allow the study of spontaneous metastatic ability are not yet available. Blinding to assess the outcome of in vivo experiments was not done. Animals of each litter were randomly allocated by age to the different experimental groups. Seven mice were enrolled in each test group in order to have an 80% chance of showing, with a 5% significance, a 55% of success in the experimental group. After 4 weeks, animals were sacrificed, and an accurate necropsy was performed. Metastatic lesions at lymph nodes, interscapular brown fat, kidneys, adrenal glands, and other sites were recorded at necropsy. Lungs, stained with black India ink to better outline metastases, and livers were fixed in Fekete’s solution. Lung and liver metastases were counted at a stereomicroscope after dissection of the organs in lobes but without dissection of each individual metastasis. Dimension of liver metastases macroscopically visible on the surface of liver lobes were measured with calipers and individual metastasis volume was calculated as π·[√(a·b)]^3^/6 where a and b are the two maximal perpendicular diameters.

### Statistical analysis

Quantitative data are described as the mean/median ± SD and were compared by Student’s *t-*test and ANOVA with Bonferroni’s post-test multiple comparisons test when appropriate. Quantitative data are described as the mean ± SD and were compared by two-tailed unpaired Student’s *t*-test and ANOVA with Dunnet’s post-test multiple comparisons test when appropriate. Mean ± SEM was used to compare *LMNA* gene expression in primary versus metastatic tumors and for individual liver metastasis volume analyses.

Correlation analysis was performed by using Spearman rank correlation analysis. Survival probability was estimated by the Kaplan-Meier method and log-rank tests were used to compare overall survival between groups according to the median value of *LMNA* gene expression.

All in vitro experiments were repeated at least three times independently with at least three replicates per experiment, and representative experiments are shown. For evaluation of in vivo results, observations were not normally distributed, therefore differences in metastasis number and volume were analyzed using the nonparametric Mann-Whitney test. Differences with *p* values <0.05 were considered statistically significant (*p* values: *p < 0.05; **p < 0.01; ***p < 0.001). Statistical analyses were performed with Graph Prism Software (version 5).

## Results

### Low expression of *LMNA* correlates with tumor aggressiveness and poor prognosis in Ewing Sarcoma

**Fig. 1:**
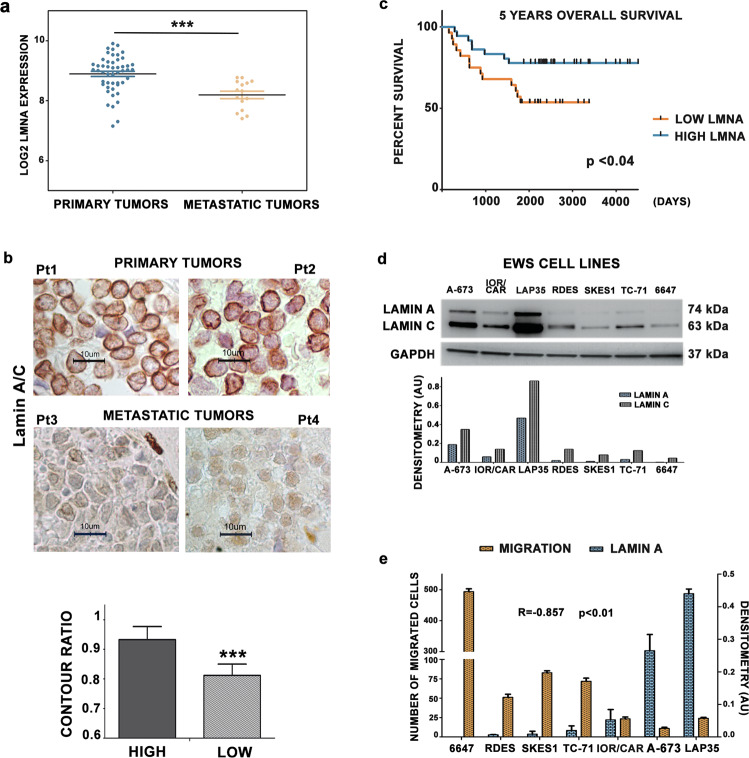
Low levels of *LMNA* correlate with poor prognosis in EWS patients. **a** *LMNA* gene expression levels (GSE17679) in EWS patients with primary tumors and metastatic tumors. Asterisks indicate statistically significant differences in *LMNA* gene expression between primary and metastatic tumors; two-tailed unpaired Student’s *t*-test, ***p < 0.001. Mean ± SEM are shown; **b** Representative images of immunohistochemical staining for lamin A in four EWS patient samples. Magnification 100X, scale bar 10 μm. Contour ratio of nuclei determined in four EWS samples with high and low *LMNA* expression, based on GSE17679 is reported in the graph. At least 100 nuclei were analyzed for each patient sample. Patients were chosen from GSE17679 (high *LMNA* R72, R80, low *LMNA* expression R29, R48). Representative patient samples are shown. Asterisks indicate statistically significant differences with respect to high lamin A sample; two-tailed unpaired Student’s t-test, ***p < 0.001; **c** Comparison between values of *LMNA* gene expression and five-years overall survival shown by Kaplan-Meier curve, *p* < 0.04; **d** Western blotting analysis of lamin A/C protein expression in EWS cell lines. Graph shows densitometric analysis as ratio referred to GAPDH, used as loading control; **e** Lamin A expression levels and migration ability values were plotted. Spearman’s rank correlation test demonstrated a significant inverse correlation among these two characteristics (**p < 0.01).

To explore the role of lamin A/C in EWS, we performed an in silico analysis exploiting microarray datasets (GSE 17679) [29]. Sixty-four EWS patients were divided into two groups according to whether the evaluation of *LMNA* transcript expression was performed on primary tumors or metastatic lesions. The comparison showed a significantly lower expression of *LMNA* gene in the metastasis (Fig. 1a). Representative clinical samples derived from tissue samples from GSE17679 patients (2 primary local tumors vs 2 metastatic lesions) with different *LMNA* gene expression, were assessed to verify the status of the shape of the cell nuclei in relation to lamin A expression levels (Fig. 1b). We performed contour ratio analysis of nuclei, finding a statistically significant difference in the contour ratio values between cells from high expressors (2 primary lesions) and cells from low expressors (2 metastatic lesions) of lamin A values (Fig. 1b).

In addition, by using another dataset (GSE63157) including clinical data from primary EWS tumors, we assessed the correlation between *LMNA* gene expression and five years overall survival of EWS patients [30]. Patients were classified as high- or low- *LMNA* expressors, according to the median value of *LMNA* gene expression. The Kaplan-Meier survival analysis indicated that patients with high expression of *LMNA* in their primary tumors had a significantly better overall survival (*p* < 0.04) (Fig. 1c).

These results indicated a role for lamin A/C in the regulation of EWS aggressiveness and prompted us to perform deeper in vitro analysis using a panel of patient-derived EWS cell lines. We measured lamin A/C protein amount and we found that it is generally expressed at low levels, except for LAP-35 cell line, which derives from a PNET tumor and shows a more differentiated phenotype [26] (Fig. 1d). Since migration ability is fundamental to support metastatic processes, we correlated lamin A/C protein expression with migration abilities of EWS cells and we found a significant inverse correlation between lamin A expression and migration (Spearman’s rank correlation *r* = -0.857; *p* < 0.01) (Fig. 1e).

### Lamin A expression significantly decreases invasiveness in EWS cells

**Fig. 2:**
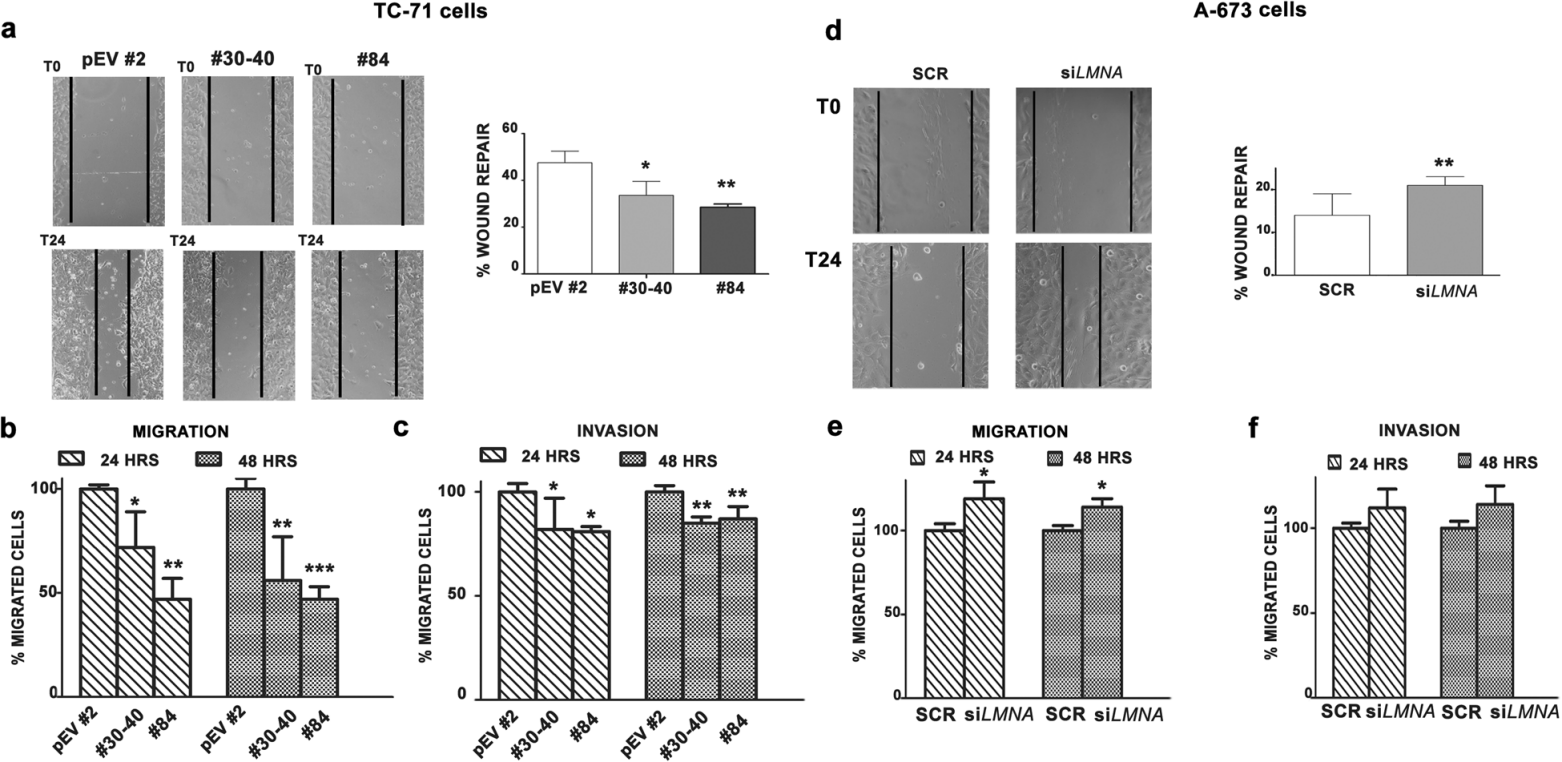
Lamin A expression influences migration and invasion abilities of EWS cells. **a** Wound healing assay of Empty-GFP clone (pEV #2), lamin A-GFP #30-40 (#30-40) and lamin A-GFP #84 (#84). Representative pictures were taken at 0 and 24 h after scratching. Magnification 10×. Histograms were plotted as mean ± SD of three independent experiments. Asterisks indicate statistically significant differences with respect to Empty-GFP clone; one-way ANOVA test, *p < 0.05, **p < 0.01; **b** Migration assay of Empty-GFP clone (pEV #2), lamin A-GFP #30-40 (#30-40) and lamin A-GFP #84 (#84) performed at 24 and 48 hours. Histograms show the percentage of migrated cells respect to Empty-GFP clone, which was considered as 100%. Histograms were plotted as mean ± SD of three independent experiments. Asterisks indicate statistically significant differences with respect to Empty-GFP clone; one-way ANOVA test, *p < 0.05, **p < 0.01, ***p < 0.001; **c** Invasion assay of Empty-GFP clone (pEV #2), lamin A-GFP #30-40 (#30-40) and lamin A-GFP #84 (#84) performed at 24 and 48 hours. Histograms show the percentage of migrated cells respect to Empty-GFP clone, which was considered as 100%. The mean ± SD of three independent experiments were plotted. Asterisks indicate statistically significant differences with respect to Empty-GFP clone; one-way ANOVA test, *p < 0.05, **p < 0.01; **d** Wound healing assay of siRNA scramble cells (SCR) and si*LMNA* A-673 (si*LMNA*). Representative pictures were taken at 0 and 24 h after scratching. Magnification 10×. Histograms were plotted as mean ± SD of three independent experiments. Asterisks indicate statistically significant differences with respect to siRNA scramble cells; two-tailed unpaired Student’s t-test, **p < 0.01; **e** Migration assay of siRNA scramble cells (SCR) and si*LMNA* A-673 (si*LMNA*) performed at 24 and 48 hours. Histograms show the percentage of migrated cells respect to siRNA scramble cells, which was considered as 100%. Histograms were plotted as mean ± SD of three independent experiments. Asterisks indicate statistically significant differences with respect to siRNA scramble cells; two-tailed unpaired Student’s t-test, *p < 0.05; **f** Invasion assay of siRNA scramble cells (SCR) and si*LMNA* A-673 (si*LMNA*) performed at 24 and 48 hours. Histograms show the percentage of migrated cells respect to siRNA scramble cells, which was considered as 100%. The mean ± SD of three independent experiments were plotted.

To understand the function of lamin A in cell migration and invasion in EWS, we took a gain- or loss-of-function approach to force or silence the expression of lamin A. Firstly, we stably expressed lamin A in TC-71 cell line (Supplementary Fig. 1a, b), while transient overexpression was obtained in the IOR/CAR cell line (Supplementary Fig. 1c). Motility and migration assays demonstrated that lamin A-expressing cells were significantly less able to migrate compared to Empty-GFP cells (Fig. 2a, b; Supplementary Fig. 1d). A significantly reduced invasion ability was also observed in TC-71 EWS transfected with lamin A compared to controls (Fig. 2c). Conversely, silencing of *LMNA* in A-673 (Supplementary Fig. 2a) or in LAP-35 (Supplementary Fig. 2b) led to a significant higher ability to repair the wound versus scramble siRNA-transfected cells (Fig. 2d; Supplementary Fig. 2d). Further, *LMNA*-silenced A-673 cells migrated significantly faster, compared to controls and were more able to invade (Fig. 2e, f).

Because it has been previously demonstrated that lamin A can be silenced by promoter hypermethylation [18, 23, 33], we also treated EWS cells with 5-Azacytidine, an inhibitor of DNA methyltransferase, confirming that lamin A/C was up-regulated both at mRNA and protein expression levels (Supplementary Fig. 3a, b) and, accordingly, cell migratory ability was significantly reduced (Supplementary Fig. 3c). Overall, these findings showed that lamin A acts as tumor suppressor and that its expression inversely correlates with invasiveness of EWS.

### Forced expression of lamin A significantly decreases metastatic load in the liver

**Fig. 3:**
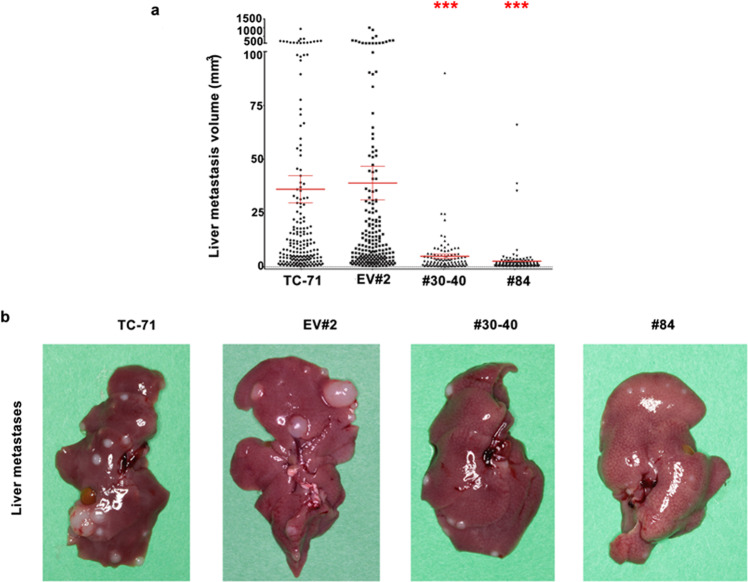
Lamin A expression decreases liver metastatic load. **a** Individual liver metastasis volume for TC-71 parental cells (*n* = 190), TC-71 EV#2 (EV#2) (*n* = 199), TC-71 lamin A-GFP #30-40 (#30-40) (*n* = 100) or TC-71 lamin A-GFP #84 (#84) (*n* = 115), red lines represent Mean ± SEM for each group. ***p < 0.001 compared to TC-71 parental cells and TC-71 EV#2 by the Mann-Whitney test; **b** Representative images of liver metastases in one mouse of each different group are shown.

As reported previously [32], most human sarcomas display a high metastatic ability in Rag2-/-;Il2rg-/- mice, which constitutively lack T, B, and NK immune response, showing a distinctive pattern of organ preference involving mainly liver, lung and other sites such as lymph nodes, interscapular brown fat pad, kidneys, and adrenal glands. This attitude was confirmed when we i.v. injected TC-71 cells and their derived stably transfected clones (Empty-GFP vector #2, lamin A-GFP #30-40 and lamin A-GFP #84) in double knockout Rag2-/-;Il2rg-/- mice. Metastases were observed in lungs, liver, lymph nodes, interscapular brown fat, kidneys, and adrenal glands. A significant difference was observed in the growth of liver metastases that was severely reduced in mice that received clones overexpressing lamin A, relative to parental TC-71 or Empty-GFP vector #2 injected animals (Fig. 3a, b and legend therein). A global although not statistically significant reduction in the total number of metastases was observed in mice after injection of TC-71 lamin A-GFP #30-40 (#30-40) or TC-71 lamin A-GFP #84 (#84) cells overexpressing lamin A (Supplementary Fig. 4a). Lung metastases in mice receiving Lamin A transfectant clones were reduced compared to parental cells but were not reduced compared to Empty-GFP vector #2 (Supplementary Fig. 4b).

### Lamin A expression rescues altered LINC complex proteins in EWS cells

Lamins, with the associated LINC complex proteins, modulate cytoskeletal structure, dynamics, and polarity, all of which are essential for cell migration [34]. In fact, alteration of nucleo-cytoskeletal coupling by disruption of the LINC-complex or lack of A-type lamins can result in altered cytoskeletal organization and altered cell polarization and migration [16, 17, 35].

**Fig. 4:**
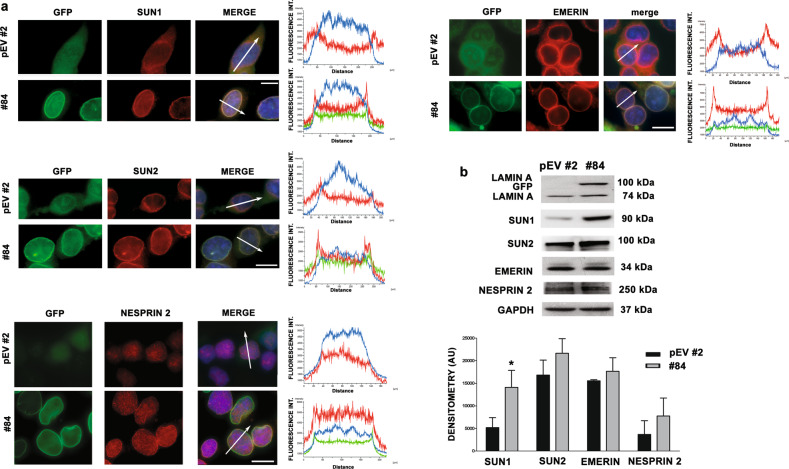
LINC complex localization in EWS cells is rescued by lamin A expression. **a** Lamin A-GFP (green), SUN1 (red), SUN2 (red), nesprin 2 (red) and emerin (red) localization in TC-71 Empty-GFP clone (pEV #2) and TC-71 lamin A-GFP #84 (#84). DNA was counterstained with 2-(4-amidinophenyl)-1H -indole-6-carboxamidine (DAPI). Merge of fluorescence signals are shown (MERGE). Graphs indicate the fluorescence intensity profile along the white arrows. Representative graphs of at least 30 nuclei analyzed for each sample were shown; magnification 100x, scale bar 10 μm; **b** Western blotting analysis of lamin A, SUN1, SUN2, emerin and nesprin 2 in TC-71 Empty-GFP clone (pEV #2) and TC-71 lamin A-GFP #84 (#84). GAPDH was used as loading control. Densitometric analysis is shown as mean values ± SD of three different experiments. Asterisks indicate statistically significant differences with respect to TC-71 Empty-GFP clone; two-tailed unpaired Student’s t-test, *p < 0.05.

In vitro, impaired anchorage of nuclear envelope proteins was observed in EWS cells. In particular, SUN1, SUN2 and nesprin 2, major LINC constituents, and emerin, the main lamin A partner protein at the nuclear envelope, were also localized to the cytoplasm, although nuclear rim anchorage was partially maintained (Fig. 4a, Supplementary Fig. 5). Of note, it has been demonstrated that mislocalization of emerin in cancer cells leads to nuclear shape instability and it is correlated to a more aggressive phenotype in in vitro and in vivo models [36]. We found that lamin A overexpression was sufficient to recruit SUN1 to the nuclear envelope (Fig. 4a), while determining an overall increase of its protein levels (Fig. 4b). Further, forced expression of lamin A in EWS cells also induced a mild increase in protein levels of SUN2 and nesprin 2, relative to naïve EWS cells (Fig. 4b), suggesting that impaired anchorage of LINC complex components is fully dependent on lamin A downregulation in EWS cells (Fig. 4a, b).

### Lamin A acts as a regulator of YAP and ROCK2 mechanosignaling effectors and induces differentiation markers in EWS

Lamin A is known to influence mechanosignaling dynamics through multiple pathways. Besides the LINC complex interplay investigated above, lamin A can also regulate nuclear import of transcriptional regulators involved in cytoskeleton remodeling. Among such effectors, we focused our attention on YAP/TAZ. Deleterious effects of lamin A deficiency on YAP/TAZ nuclear import have been recently demonstrated in muscle cells [37, 38]. Interestingly, YAP nuclear import upon mechanical strain was favored by non-functional lamin A/C and it was associated with increased YAP protein levels [38]. Most importantly, a recent work showed a direct correlation between YAP/TAZ levels and activity and metastatic potential in EWS [39].

**Fig. 5:**
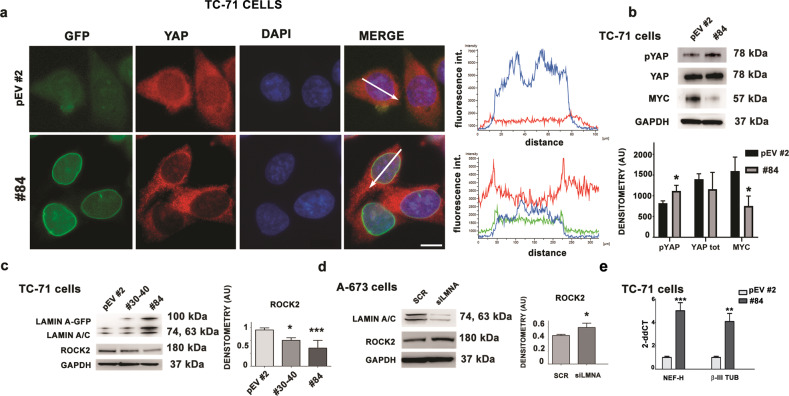
The expression of lamin A affects YAP and ROCK2 activity and stimulates neural differentiation in EWS cells. **a** Lamin A-GFP (green) and YAP (red) localization in Empty-GFP clone (pEV #2) and lamin A-GFP #84 (#84). DNA was counterstained with DAPI (DAPI). Merge of fluorescence signals are shown (MERGE). Graphs indicate the fluorescence intensity profile along the white arrows. Representative graphs of at least 30 nuclei analyzed for each sample were shown; magnification 100x, scale bar 10 μm; **b** Western blotting analysis of YAP, p(Ser127) YAP and MYC protein expression in Empty-GFP clone (pEV #2) and lamin A-GFP #84 (#84). GAPDH was used as loading control. Densitometric analysis is shown as mean values ± SD of three different experiments. Asterisks indicate statistically significant differences with respect to Empty-GFP clone; two-tailed unpaired Student’s t-test, *p < 0.05; **c** Western blotting analyses of lamin A/C and ROCK2 protein expression in Empty-GFP clone (pEV #2), lamin A-GFP #30-40 (#30-40) and lamin A-GFP #84 (#84). GAPDH was used as loading control. Densitometric analysis is shown as mean values ± SD of three different experiments. Asterisks indicate statistically significant differences with respect to Empty-GFP clone; one-way ANOVA test, *p < 0.05, ***p < 0.001; **d** Western blotting analysis of lamin A/C and ROCK2 protein expression in siRNA scramble cells (SCR) and *siLMNA* A-673 (*siLMNA*). GAPDH was used as loading control. Densitometric analysis is shown as mean values ± SD of three different experiments. Asterisks indicate statistically significant differences with respect to siRNA scramble cells; two-tailed unpaired Student’s t-test, *p < 0.05; **e** qRT-PCR analysis of *NEF-H* and β*3-tubulin* in Empty-GFP clone (pEV #2) and lamin A-GFP #84 (#84). Data are shown as 2-^ΔΔ*Ct*^. GAPDH was used as a housekeeping gene^.^ Data are shown as mean values ± SD of three different experiments. Asterisks indicate statistically significant differences with respect to Empty-GFP clone; two-tailed unpaired Student’s t-test, **p < 0.01; ***p < 0.001.

In EWS cells, we observed YAP recruitment to the nucleoplasm, while the overexpression of lamin A reduced its nuclear retention and increased its phosphorylation (Fig. 5a, b). Moreover, in EWS cells overexpressing lamin A, significant downregulation of MYC, previously identified as a decisive target of YAP, was measured (Fig. 5b).

Due to the role of ROCK2 kinase (Rho-associated protein kinase 2) as a crucial driver of EWS cell migration trough regulation of the actin cytoskeleton and cell movement [40], we also evaluated ROCK2 expression by western blotting analysis, and found a significant reduction of ROCK2 protein levels in lamin A overexpressing cells, compared to Empty-GFP cells (Fig. 5c). On the contrary, western blotting analysis showed upregulation of ROCK2 in *siLMNA* transfected cells (Fig. 5d). All in all, these results supported a key role of lamin A in EWS as regulator of YAP and ROCK2 mechanosignaling effectors related to invasiveness (Fig. 5). Moreover, TC-71 overexpressing lamin A (clone #84) showed significantly increased expression of neural markers, including *NEF-H* and *β3-tubulin* relative to TC-71 empty vector cells (Fig. 5e), which suggests activation of neural differentiation.

### Prelamin A accumulation decreases migration and invasion abilities of EWS cells

**Fig. 6:**
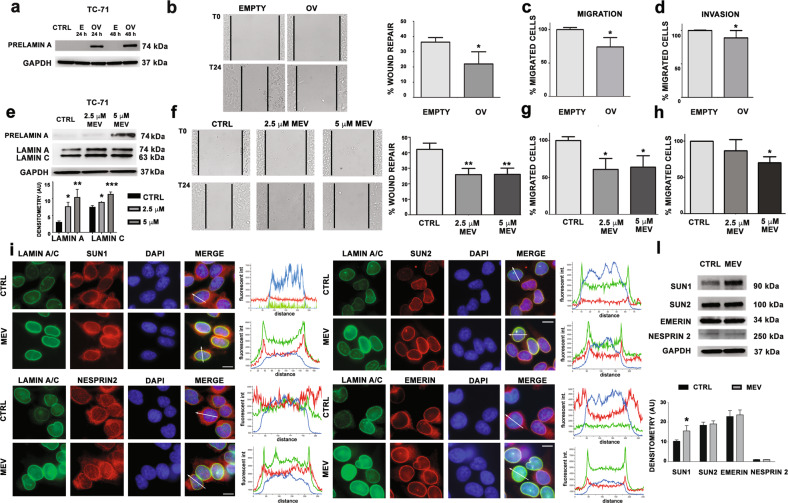
Prelamin A-related decrease of cell migration and motility in EWS cells. **a** Western blotting analysis of prelamin protein expression in parental TC-71 (CTRL), empty vector cells (E) and prelamin transfected TC-71 cells (OV) performed at 24 and 48 hours. GAPDH was used as loading control; **b** Wound healing assay of empty vector cells (EMPTY) and prelamin overexpressed TC-71 cells (OV). Representative pictures were taken at 0 and 24 h after scratching. Magnification 10×. Histograms were plotted as mean ± SD of three independent experiments. Asterisks indicate statistically significant differences with respect to empty vector cells; two-tailed unpaired Student’s t-test, *p < 0.05; **c** Migration assay of empty vector cells (EMPTY) and prelamin overexpressed TC-71 cells (OV). Histograms show the percentage of migrated cells respect to empty vector cells, which were considered as 100%. Histograms were plotted as mean ± SD of three independent experiments. Asterisks indicate statistically significant differences with respect to empty vector cells; two-tailed unpaired Student’s t-test, *p < 0.05; **d** Invasion assay of empty vector cells (EMPTY) and prelamin overexpressed TC-71 cells (OV). Histograms show the percentage of migrated cells respect to empty vector cells, which were considered as 100%. Histograms were plotted as mean ± SD of three independent experiments. Asterisks indicate statistically significant differences with respect to empty vector cells; two-tailed unpaired Student’s t-test, *p < 0.05. **e** Western blotting analysis of prelamin A and lamin A/C protein expression in non-treated TC-71 cells (CTRL) and in mevinolin treated EWS cells (2.5 μM or 5μM MEV). GAPDH was used as loading control. Densitometric analyses are shown as mean values ± SD of three different experiments. Asterisks indicate statistically significant differences with respect to CTRL cells; one-way ANOVA test, *p < 0.05, **p < 0.01, ***p < 0.001; **f** Wound healing assay of non-treated TC-71 cells (CTRL) and mevinolin treated EWS cells (2.5 μM or 5μM MEV). Representative pictures were taken at 0 and 24 h after scratching. Magnification 10×. Histograms were plotted as mean ± SD of three independent experiments. Asterisks indicate statistically significant differences with respect to non-treated TC-71 cells; one-way ANOVA test, **p < 0.01; **g** Migration assay of non-treated TC-71 cells (CTRL) and mevinolin treated EWS cells (2.5 μM or 5μM MEV). Histograms show the percentage of migrated cells respect to non-treated TC-71 cells, which were considered as 100%. Histograms were plotted as mean ± SD of three independent experiments. Asterisks indicate statistically significant differences with respect to non-treated TC-71 cells; one-way ANOVA test, *p < 0.05; **h** Invasion assay of non-treated TC-71 cells (CTRL) and mevinolin treated EWS cells (2.5 μM or 5μM MEV). Histograms show the percentage of migrated cells respect to non-treated TC-71 cells, which were considered as 100%. Histograms were plotted as mean ± SD of three independent experiments. Asterisks indicate statistically significant differences with respect to non-treated TC-71 cells; one-way ANOVA test, *p < 0.05. **i** Lamin A/C (green), SUN1 (red), SUN2 (red), Nesprin2 (red), and Emerin (red) localization in non-treated TC-71 cells (CTRL) and mevinolin treated (5 μM) EWS cells (MEV). DNA was counterstained with DAPI (DAPI). Merge of fluorescence signals are shown (MERGE). Graphs indicate the fluorescence intensity profile along the white arrows. Representative graphs of at least 30 nuclei analyzed for each sample were shown; magnification 100x, scale bar 10 μm; **l** Western blotting analysis of SUN1, SUN2, Emerin, and Nesprin 2 in non-treated TC-71 cells (CTRL) and mevinolin treated (5 μM) EWS cells (MEV). GAPDH was used as loading control. Densitometric analysis is shown as mean values ± SD of three different experiments. Asterisks indicate statistically significant differences with respect to CTRL cells; two-tailed unpaired Student’s t-test, *p < 0.05.

Prelamin A, the precursor protein of lamin A, plays a pivotal role in chromatin organization and in transcriptional regulation. While extremely low prelamin A levels are detected in normal cells due to rapid protein maturation, moderate accumulation of prelamin A has been reported to decrease the invasion potential of tumor cells, both in cellular models [41] and laminopathic mice [42]. We wanted to evaluate if prelamin A accumulation could reduce the invasiveness of EWS cells. To test this hypothesis, we expressed a mutated prelamin A sequence, which causes the accumulation of non-farnesylated prelamin A (LA-C661M) [13]. Western blot analysis showed accumulation of prelamin A in transfected EWS cells (Fig. 6a). Interestingly, a significant decrease in cellular motility, migration and invasion abilities was measured in prelamin A overexpressing EWS cells (Fig. 6b–d).

Based on the latter finding, we induced prelamin A accumulation by a pharmacological approach. To this end, we employed mevinolin, that, as all statins, inhibits the mevalonate pathway and farnesyl production, which is necessary for prelamin A farnesylation. Treatment with mevinolin causes accumulation of non-farnesylated prelamin A, which cannot undergo protein maturation steps [13, 16]. Interestingly, we found that mevinolin treatment not only induced prelamin A accumulation, as expected, but also caused a significant increase in lamin A/C protein levels (Fig. 6e) and this was accompanied by a reduction in EWS cell migration and invasion abilities (Fig. 6f–h). Moreover, mevinolin treatment was able to rescue localization of LINC complex proteins, accompanied by an increase in SUN1 protein levels, as observed in EWS cells overexpressing lamin A (Fig. 6i, l and Supplementary Fig. 5).

### Mevinolin drives differentiation of EWS cells and rescues mechanosignaling effectors

**Fig. 7:**
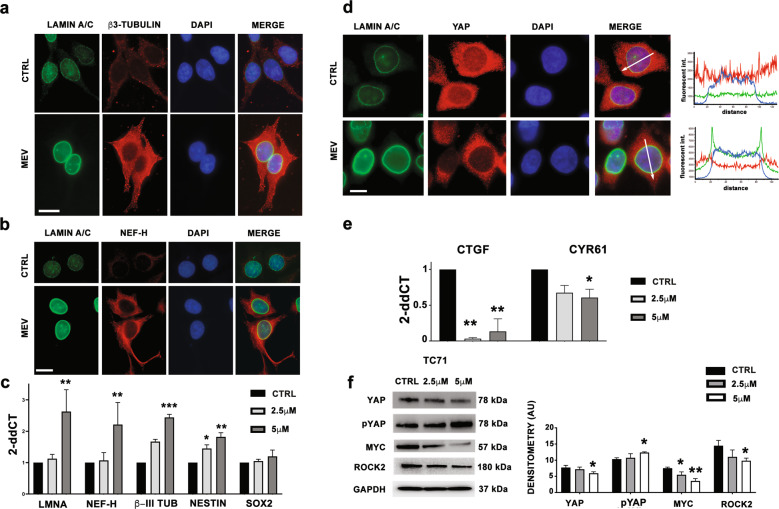
Mevinolin induces neural differentiation and rescues YAP and ROCK2 dynamics in EWS cells. **a** Lamin A/C (green) and β3-tubulin (red) localization in non-treated TC-71 cells (CTRL) and in mevinolin treated (5 μM) EWS cells (MEV). DNA was counterstained with DAPI (DAPI). Merge of fluorescence signals are shown (MERGE); magnification 100x, scale bar 10 μm; **b** Lamin A/C (green) and neurofilament-H (NEF-H) (red) localization in non-treated TC-71 cells (CTRL) and in mevinolin treated (5 μM) EWS cells (MEV). DNA was counterstained with DAPI (DAPI). Merge of fluorescence signals are shown (MERGE); magnification 100x, scale bar 10 μm; **c** qRT-PCR analysis of *LMNA*, *NEF-H*, β3-tubulin, nestin and *SOX2* genes in untreated TC-71 cells (CTRL) and in mevinolin treated EWS cells (2.5 μM or 5μM MEV). Data are shown as 2-^ΔΔ*Ct*^. GAPDH was used as a housekeeping gene. Data are shown as mean values ± SD of three different experiments. Asterisks indicate statistically significant differences with respect to CTRL cells; one-way ANOVA test, *p < 0.05, **p < 0.01, ***p < 0.001; **d** Lamin A/C (green) and YAP (red) localization in non-treated TC-71 cells (CTRL) and in mevinolin treated (5 μM) EWS cells (MEV). DNA was counterstained with DAPI (DAPI). Merge of fluorescence signals are shown (MERGE). Graphs indicate the fluorescence intensity profile along the white arrows. Representative graphs of at least 30 nuclei analyzed for each sample were shown; magnification 100x, scale bar 10 μm; **e** qRT-PCR analysis of *CTGF* and *CYR61* in non-treated TC-71 cells (CTRL) and in mevinolin treated EWS cells (2.5 μM or 5μM MEV). Data are shown as 2-^ΔΔ*Ct*^. GAPDH was used as a housekeeping gene. Data are shown as mean values ± SD of three different experiments. Asterisks indicate statistically significant differences with respect to CTRL cells; one-way ANOVA test, *p < 0.05, **p < 0.01; **f** Western blotting analysis of YAP, p(Ser127) YAP, MYC and ROCK2 protein expression in non-treated TC-71 cells (CTRL) and in mevinolin treated EWS cells (2.5 μM or 5μM MEV). GAPDH was used as loading control. Densitometric analysis is shown as mean values ± SD of three different experiments. Asterisks indicate statistically significant differences with respect to CTRL cells; one-way ANOVA test, *p < 0.05, **p < 0.01.

Thus, mevinolin treatment induced upregulation of lamin A/C, along with the expected accumulation of prelamin A. Lamin A upregulation can elicit two different but strictly interconnected pathways. On the one side, modulation of lamin A levels is linked to differentiation [18]; on the other side, it drives cytoskeleton remodeling [43–45]. Both these phenomena were observed upon mevinolin treatment in EWS cells. In fact, mevinolininduced accumulation of neural differentiation protein markers, such as β3-tubulin and neurofilament-H, which are expressed at the early stages of neuronal development (Fig. 7a, b). Upregulation of *nestin*, *β3-tubulin* and *NEF*-*H* genes, associated with a significant increase in *LMNA* gene expression, was further shown by qRT-PCR analysis (Fig. 7c). We also evaluated *SOX2* gene expression levels, due to its critical role in EWS [46, 47]. Indeed, *SOX2* is one of the targets of EWS/FLI, and it has been identified as an oncogenic factor in processes associated with tumor progression, including cell proliferation and tumorigenesis of Ewing Sarcoma family tumors [48]. *SOX2* gene expression was unaffected by the treatment with mevinolin (Fig. 7c). To assess whether lamin A/C is increased during spontaneous neural differentiation of EWS cells, we cultured TC-71 cells in low-serum medium to induce differentiation towards the neuronal lineage (Supplementary Fig. 6). Lamin A/C levels were increased in differentiating TC-71 cells, showing that lamin A/C upregulation is linked to induction of EWS differentiation (Supplementary Fig. 6).

Regarding cytoskeleton remodeling, mevinolin, not only induced the intermediate filament proteins nestin and neurofilament-H and the microtubule constituent β3-tubulin, as reported above, but also altered mechanosignaling effectors. In fact, in mevinolin-treated EWS cells, we could demonstrate downregulation of YAP/TAZ signaling and exclusion of YAP from the nucleus (Fig. 7d), as previously observed downstream of lamin A [38]. The decreased activity of YAP/TAZ signaling was demonstrated by downregulation of *CTGF* and *CYR61*, the main effectors of this pathway (Fig. 7e) [49–51], as well as by the significantly increased levels of phospho-(Ser127)-YAP, which represents the inactive form of this protein (Fig. 7f) [52]. YAP exerts its control on migration, at least in part, through the transcriptional regulation of the *CTGF* and *CYR61* promoters. These genes, which belong to the CCN (CYR61, CTGF and Nephroblastoma overexpressed gene) family, promote the epithelial-mesenchymal transition (EMT) process, allowing cancer cells to migrate and to disseminate to distant organs [53–56].

Reduced YAP/TAZ activity was further confirmed by downregulation of MYC, as YAP/TAZ is known to activate MYC at transcriptional and post-transcriptional level, promoting tumorigenesis [57]. Moreover, ROCK2 protein levels were significantly downregulated in mevinolin-treated EWS cells (Fig. 7f).

As a whole, these results show that mevinolin is able to increase lamin A and prelamin A levels, rescuing LINC complex and soluble mechanosignaling effectors YAP and ROCK2, while also inducing neural differentiation markers in EWS cells. Downstream of these events, mevinolin treatment reduces migration ability of EWS cells.

## Discussion

Discovering pathways involved in EWS metastatic process is urgently needed for designing novel and more effective therapeutic strategies. Several studies demonstrated the implication of lamins in constraining cell migration potential in different type of cancers, but their role still remains elusive in EWS [58]. Lamin A’s anti-tumorigenic activity has been reported in several human tumors as endometrial cancer or breast cancer [11, 59, 60]. Interestingly, loss of lamin A has been linked to altered protection of stalled replication forks due to defective recruitment of DNA replication factors [60]. Moreover, confined migration of tumor cells has been linked to downregulation of lamin A and increased nuclear deformability, a condition leading to high metastatic potential (Bell et al. BioRxiv 2021) [61].

Our study investigated the role of lamin A in EWS, showing a significant inverse correlation between lamin A expression and tumor invasiveness in EWS patients. In fact, our in-silico analysis showed significantly lower *LMNA* gene expression in metastatic tissue relative to primary tumors, as well as an inverse correlation between *LMNA* expression and 5-year EWS patient survival. Moreover, severe nuclear misshaping was observed in metastatic tissues, associated with almost undetectable lamin A in nuclei. Consistently, we found that overexpression of lamin A reduced motility and invasiveness of EWS cells in vitro and metastatic burden in vivo, while low levels of lamin A were associated with increased migration abilities. In vivo, the impact of lamin A was particularly evident against liver metastasis that resulted significantly smaller, suggesting that liver-produced chemoattractants, growth and motility factors such as insulin-like growth factor (IGF) and hepatocyte growth factor/scatter factor (HGF/SF), which were implicated as major molecular mechanisms of liver metastasis of sarcomas [32] may be at least partly responsible for the different behaviors of cells with high or low expression of lamin A. We further showed that prelamin A overexpression or drug-induced prelamin A accumulation also reduce EWS cell migration ability, in agreement with previous data showing that prelamin A is involved in preventing tumor cell migration and metastatic potential in oral cancers and laminopathic mice [42].

To gain mechanistic insights into lamin A-dependent pathways in EWS, we focused our study on proteins of the LINC complex, that are major lamin A partners in mechanosignaling transduction, thereby influencing cytoskeleton dynamics and cell migration [35], two major dysregulated mechanisms in cancer [62]. Indeed, the LINC complex plays pivotal and specific roles in controlling actin dynamics and nuclear positioning during cell migration and adhesion [16, 17, 63–66].

Our findings demonstrate that SUN1, nesprin2, and emerin localization in the nuclear envelope of EWS cells is affected by a lamin A-dependent mechanism. These results are consistent with previously reported data showing involvement of the LINC complex and nuclear envelope proteins, including emerin, in cancer progression and metastatization [9, 36, 58, 67]. The effects of SUN1 and SUN2 on cytoskeleton remodeling, stress fiber formation, and cell motility are mediated by LINC complex interactions with cytoskeleton components, but also rely on LINC interplay with RhoA-ROCK2 signaling machinery [63, 68, 69]. In fact, SUN1 inhibits RhoA activation and focal adhesion assembly, antagonizing SUN2 activity, which physiologically triggers the RhoA pathway [63]. Consistent with this interplay, low levels of SUN1 favor migration of bone marrow mesenchymal stem cells [68], while the predominant SUN1 splice isoform (SUN1_916) has an inhibitory effect on cellular migration in HeLa cells [69].

Here, we found that rescue of SUN1 levels and localization, either in lamin A overexpressing cells or in mevinolin-treated EWS cells, was associated with significantly lower ROCK2 levels and reduced cellular motility, in agreement with the critical role played by ROCK2 on EWS cell migration and growth [40]. Lamin A overexpression further inhibited YAP/TAZ signaling in EWS cells, which might also explain the reduced cell migration, as inhibition of the YAP/TAZ/TEAD complex with verteporfin resulted in reduced cell migration of EWS cells in vitro and verteporfin decreased metastasis formation in EWS xenograft models [39]. Inhibition of YAP/TAZ signaling was also observed in mevinolin-treated EWS cells. It has been demonstrated that the mevalonate pathway promotes YAP/TAZ nuclear localization and activity, while statins, by inhibiting HMG-CoA reductase, the rate-limiting enzyme of this pathway, lead to YAP/TAZ cytoplasmic localization and block its transcriptional responses [52]. Thus, mevinolin could act as an inhibitor of EWS cell migration by increasing prelamin A levels as well as by reducing mevalonate-dependent YAP/TAZ activity.

On the other hand, mevinolin inhibits the farnesylation of signaling effectors, including Ras and Rho, thereby reducing their activity on tumor growth and cancer cell migration. Thus, through inhibition of the mevalonate pathway, mevinolin elicits prelamin A accumulation, dampens mevalonate-dependent YAP/TAZ activity and inhibits Rho GTPase and its effector ROCK2. All these events ultimately reduce the migration ability of EWS cells. Of note, ROCK2 promotes YAP activity, whereas ROCK2 deprivation leads to the inhibition of metastatic potential in osteosarcoma cells through modulation of YAP activity [70]. Most importantly, YAP signaling is described as a prognostic marker in EWS patients as the study on 55 primary EWS samples revealed that high YAP/TAZ expression is associated with disease progression and predicts poorer outcome [71].

Upon mevinolin treatment of EWS cells, we also found an unexpected increase in mature lamin A levels. The latter finding might be related to gain of a more differentiated cellular phenotype, which is associated with lamin A expression [18, 72]. In fact, our results show that mevinolin treatment prompts EWS cells toward a more differentiated status, as demonstrated by significantly increased levels of neural markers, also in line with the fact that the downregulation of YAP activity was associated with neural differentiation [73]. EWS is a very undifferentiated tumor likely due to overexpression of CD99 protein, which prevents terminal neural differentiation [27, 74], and EWS-FLI1, which induces aberrant cell differentiation. The effect of mevinolin suggests a role for lamin A in addressing cells toward a more differentiated state, as further supported by induction of differentiation markers in EWS clones overexpressing lamin A, despite the presence and activity of either CD99 or EWS-FLI1. Interestingly, prelamin A-related effects of mevinolin and other farnesylation inhibitors (farnesyl-transferase inhibitors, FTIs) on cell differentiation have been reported in several cell types, including osteoclasts and osteoblasts [75, 76] and warrant further studies in EWS. As a whole, in mevinolin-treated EWS cells, we show prelamin A accumulation and upregulation of lamin A/C, rescue of the physiological nucleo-cytoskeleton coupling through the LINC complex, inhibition of mechanosignaling effectors ROCK2 and YAP [77, 78], and neural differentiation-related cytoskeleton remodeling. Reduced migration ability of mevinolin-treated tumor cells is the main benefit of this rescue mechanism.

**Fig. 8:**
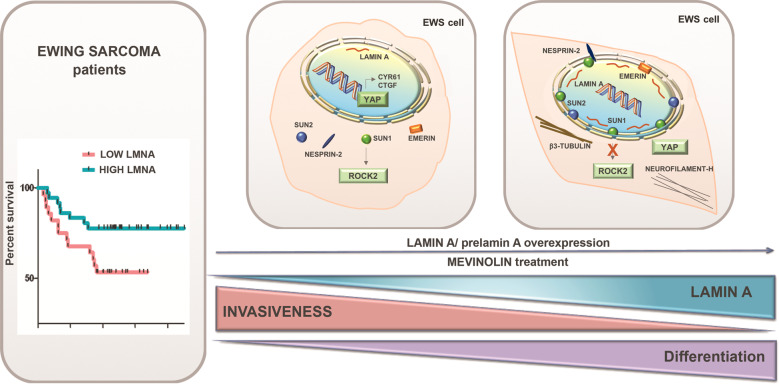
Lamin A and the LINC complex act as tumor suppressors in Ewing Sarcoma. In EWS, *LMNA* gene expression is correlated with patient survival, inversely correlated with tumor cell invasiveness and directly correlated with cellular differentiation. In EWS cells, we show displacement of LINC complex components and emerin in the cytoplasm, YAP retention in the nucleus and ROCK2 activation. Increase of prelamin A and lamin A in the nuclear lamina using either a molecular approach or mevinolin treatment rescues nuclear envelope functional organization and mechanosignaling effectors thus triggering cytoskeletal markers of neural differentiation.

Taking together, this study characterizes new lamin A-related mechanisms involved in the metastatization process of EWS cells and paves the way to other intriguing pathogenetic hypotheses for EWS based on lamin A/C interactions on the nucleoplasmic side, i.e. with chromatin and chromatin-associated proteins involved in genome organization and integrity [20, 79] (Fig. 8). Moreover, our results identify a drug, already employed in clinical practice, as a tool capable of reducing migration and invasion ability of EWS cells, while triggering neural differentiation. Mevinolin effects, which are in part due to modulation of prelamin A post-translational maturation, lamin A/C upregulation and anchorage of the LINC components, make this drug a potential candidate to be translated into clinic, especially for those EWS cases with metastatic disease at onset.

## Supplementary information

Supplementary figures with legends

aj-checklist Chiarini-CCDIS-21-2545R-2

Western blots all figures

## Data Availability

Data are available upon request.
