# Supplementary material for: Lamin A and the LINC complex act as potential tumor suppressors in Ewing Sarcoma
Source: Cell Death Dis. 2022 Apr 14;13(4):346. doi: 10.1038/s41419-022-04729-5 (PMC9010457; doi:10.1038/s41419-022-04729-5)
Supplement: Supplementary file 1 — Supplementary figures with legends [file 41419_2022_4729_MOESM1_ESM.pdf]

LAMIN A AND THE LINC COMPLEX ACT AS APOTENTIAL TUMOR SUPPRESSORS IN EWING SARCOMA

Francesca Chiarini[1,2#*], Francesca Paganelli[1,3#], Tommaso Balestra[4,5], Cristina Capanni[1,2], Antonietta Fazio[3], Maria Cristina Manara[4], Lorena Landuzzi[4] Stefania Petrini[6], Camilla Evangelisti[3], Pier-Luigi Lollini[5], Alberto M. Martelli [3], Giovanna Lattanzi[1,2*], and Katia Scotlandi[4*]

[1] CNR-Institute of Molecular Genetics "Luigi Luca Cavalli-Sforza", Unit of Bologna, 40136 Bologna, Italy

[2] IRCCS Istituto Ortopedico Rizzoli, 40136 Bologna, Italy

[3]Alma Mater Studiorum, University of Bologna, Department of Biomedical and Neuromotor Sciences, 40136 Bologna, Italy

[4] IRCCS Istituto Ortopedico Rizzoli, Experimental Oncology Laboratory, 40136 Bologna, Italy

[5]Alma Mater Studiorum, University of Bologna, Department of Experimental, Diagnostic and Specialty Medicine, 40138 Bologna, Italy

[6]Confocal Microscopy Core Facility, Research Center, Bambino Gesu' Children's Hospital IRCCS, 00146 Rome, Italy

[#] equally contributed to this work

***Correspondence to:**

giovanna.lattanzi@cnr.it (G.L.); Tel. +39-0516366857

francesca.chiarini@cnr.it (F.C.); Tel. +39-0512091582

katia.scotlandi@ior.it (K.S.); Tel +39-0516366760

Supplementary material

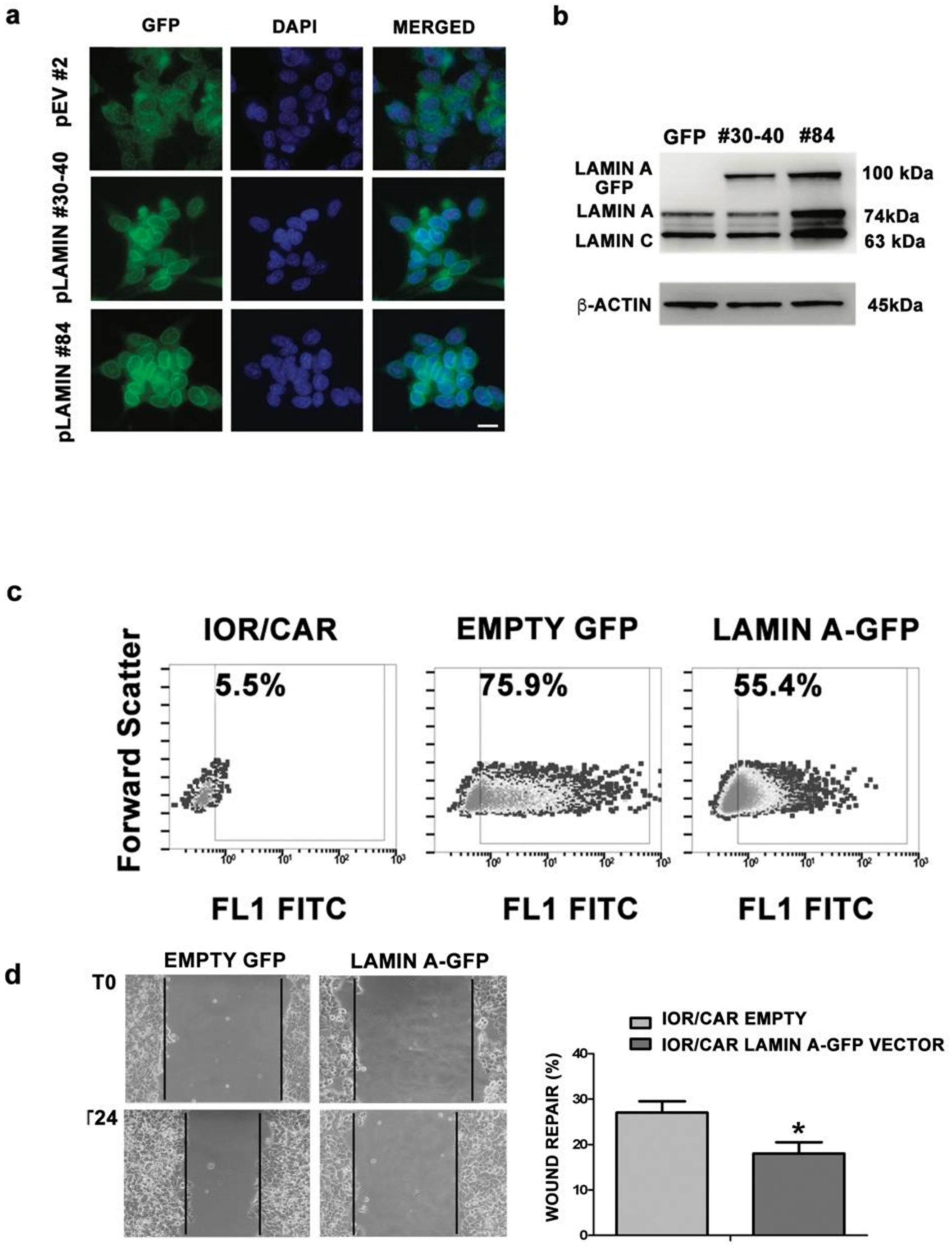

Supplementary figure 1: Overexpression of lamin A reduces migration and motility in EWS cells. (**a**) Lamin A (GFP) (green) in Empty-GFP clone (pEV #2), lamin A-GFP #30-40 and lamin A-GFP #84. DNA was counterstained with DAPI (DAPI). Merge of fluorescence signals are shown (MERGE); (**b**) Western blotting analyses of lamin A/C and lamin A/GFP protein expression in Empty-GFP clone (GFP), lamin A-GFP #30-40 (#30-40) and lamin A-GFP #84 (#84). β-actin was used as loading control; (**c**) Flow cytometric analyses of IOR/CAR EWS cells transfected with empty GFP

vector or lamin A-GFP vector (IOR/CAR parental cell line was employed to select GFP positive cells); (**d**) Wound healing assays of IOR/CAR EWS cells transfected with empty GFP vector or lamin A-GFP vector. Representative pictures were taken at 0 and 24 h after scratching. Magnification 10×. Histograms were plotted as mean ± SD of three independent experiments. Asterisks indicate statistically significant differences with respect to empty GFP transfected cells; two-tailed unpaired Student's t-test *p<0.05.

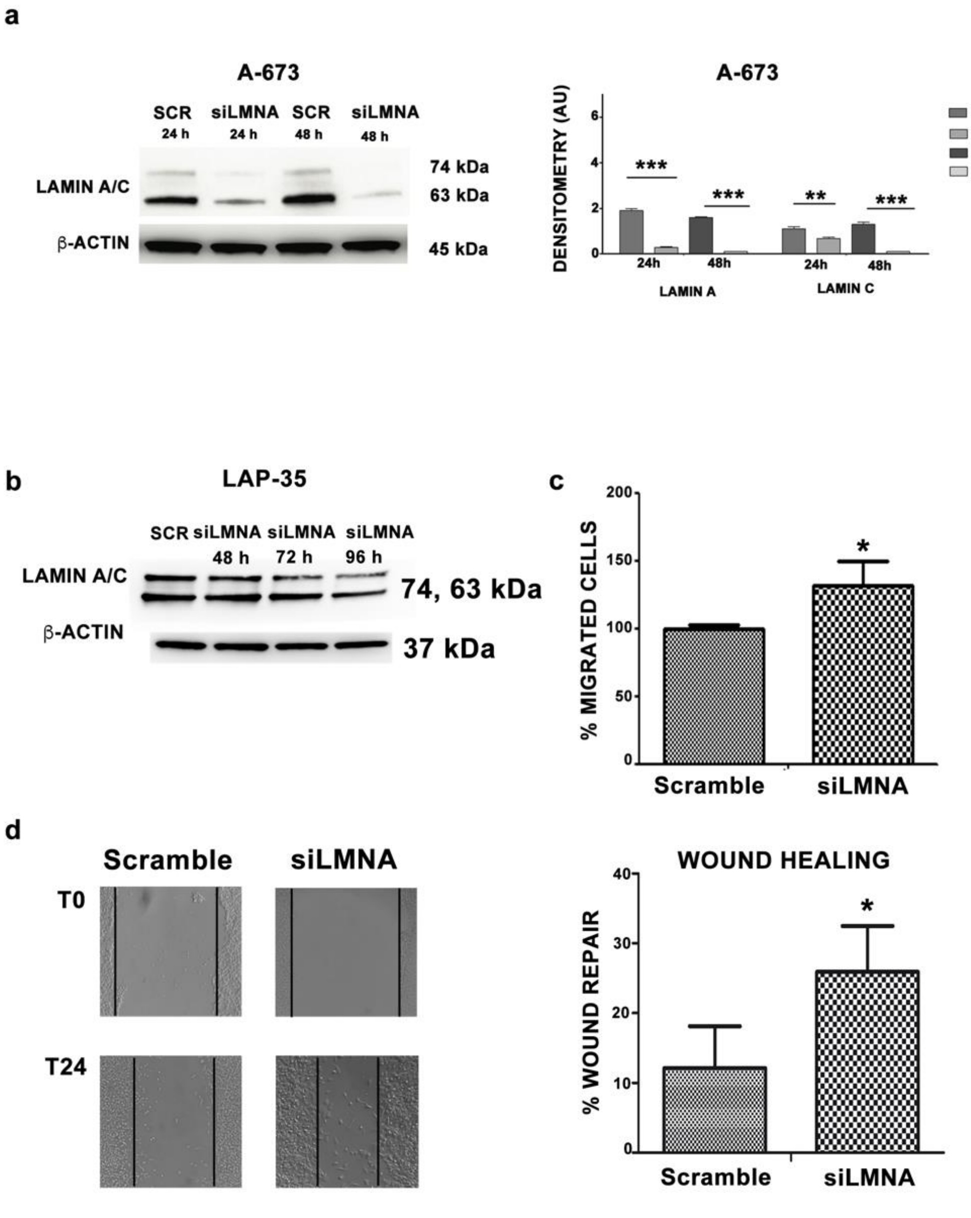

Supplementary figure 2: **Silencing of lamin A increases migration in EWS cells** (**a**) Western blotting analysis of lamin A/C protein expression A-673 siRNA scramble cells (SCR) and si*LMNA* A-673 (si*LMNA*) performed at 24 and 48 hours. β-actin was used as loading control; densitometric analysis is shown as mean values ± SD of three different experiments. Asterisks indicate statistically significant differences with respect to siRNA scramble cells at 24 or 48 hours; two-tailed unpaired Student's t-test **p<0.01, ***p<0.001; **(b)** Western blotting analysis of lamin A/C protein expression in LAP-35 siRNA scramble cells (SCR) and si*LMNA* LAP-35 (si*LMNA*) performed at 48, 72, 96 hours. β-actin was used as

loading control. (**c**) Migration assay of siRNA scramble cells (SCR) and si*LMNA* LAP-35 (si*LMNA*) performed at 24 hours. Histograms show the percentage of migrated cells with respect to siRNA scramble cells, which was considered as 100%. Histograms were plotted as mean ± SD of three independent experiments. Asterisks indicate statistically significant differences with respect to siRNA scramble cells; two-tailed unpaired Student's t-test, *p<0.05; (**d)** Wound healing assay of siRNA scramble cells (SCR) and si*LMNA* LAP-35 (si*LMNA*). Representative pictures were taken at 0 and 24 h after scratching. Magnification 10×. Histograms were plotted as mean ± SD of three independent experiments. Asterisks indicate statistically significant differences with respect to siRNA scramble cells; two-tailed unpaired Student's t-test, *p<0.05.

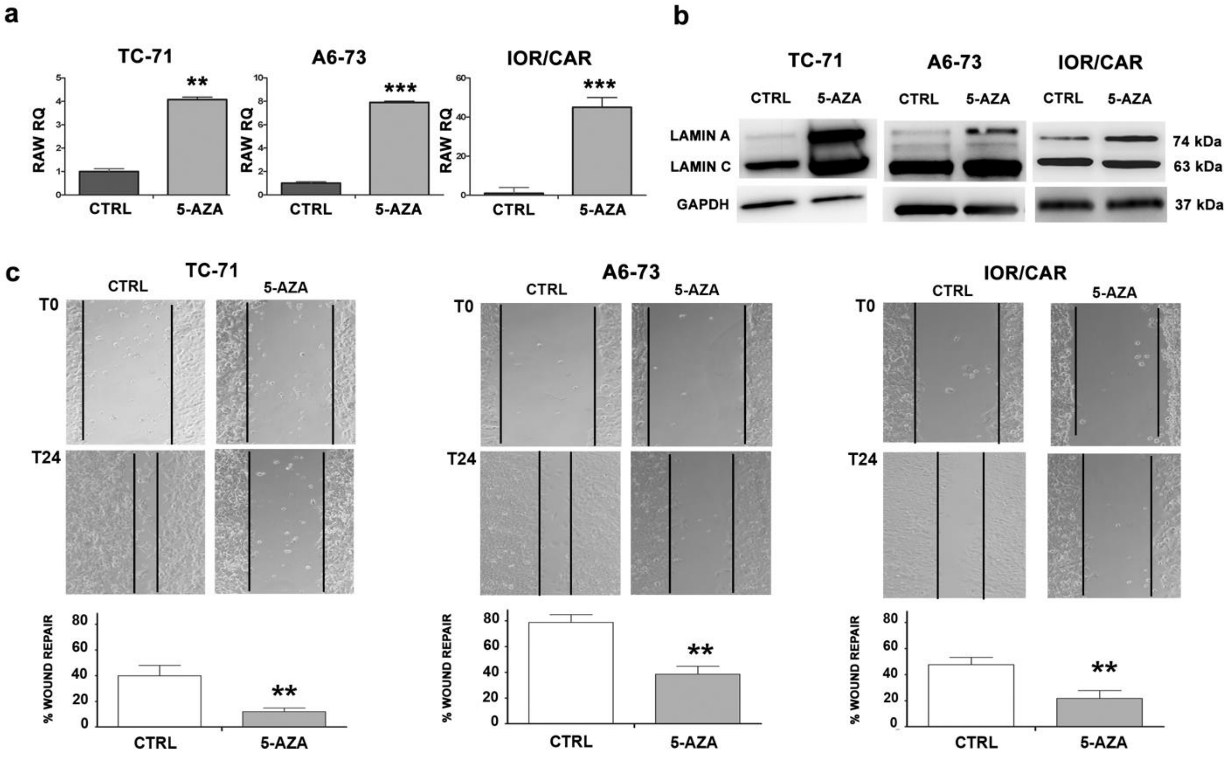

Supplementary figure 3: **5-Azacytidine significantly reduces motility in EWS cells. (a)** Increased *LMNA* gene expression by qRT-PCR analysis after 48 hours of treatment with 5-Azacytidine (4 μM). Asterisks indicate statistically significant differences with respect to not-treated EWS cells (CTRL). Histograms were plotted as mean ± SD of three independent experiments; two-tailed unpaired Student's t-test **p< 0.01, ***p< 0.001; (**b**) Western blotting analysis of lamin A/C protein expression in TC-71, A-673 and IOR/CAR cell lines treated with 5-Azacytidine (4 μM). GAPDH was used as loading control; (**c**) Wound healing assay of TC-71, A-673 and IOR/CAR. Wound repair capacity was evaluated in non-treated EWS cell lines (CTRL) versus 5-Azacytidine treated EWS cells (5-AZA). Representative pictures were taken at 0 and 24 h after scratching. Magnification 10×. Histograms were plotted as mean ± SD of three independent experiments. Asterisks indicate statistically significant differences with respect to CTRL EWS cells; two-tailed unpaired Student's t-test, **p<0.01.

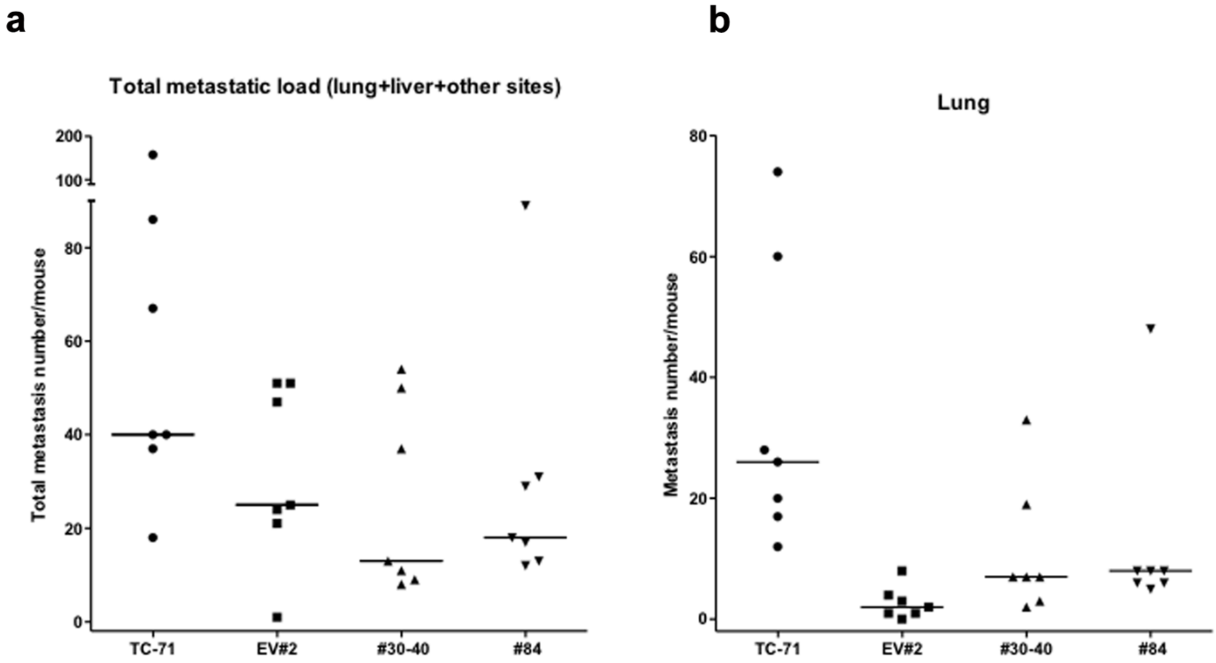

Supplementary figure 4: Total metastatic load and lung metastatic load in mice receiving i.v. injection of TC-71 cells and empty vector or lamin A transfectant clones. (a) Total metastatic load in mice receiving i.v. injection of TC-71 cells, Empty-GFP clone (pEV #2), lamin A-GFP #30-40 clone (#30-40) and lamin A-GFP #84 clone (#84), expressed as total number of metastasis per mouse including metastasis to the lung, liver and other sites such as lymph nodes, interscapular brown fat, kidneys and adrenal glands. Black lines represent the median number of metastases for each group. $p<0.05$ for #84 compared to TC-71 parental cells by the Mann-Whitney test. (b) Number of lung metastasis per mouse receiving i.v. injection of TC-71 cells, Empty-GFP clone (pEV #2), lamin A-GFP #30-40 clone (#30-40) and lamin A-GFP #84 clone (#84). Black lines represent the median number of metastases for each group. All of the transfectant clones were significantly different compared to TC-71 parental cells ($p<0.05$) by the Mann-Whitney test.

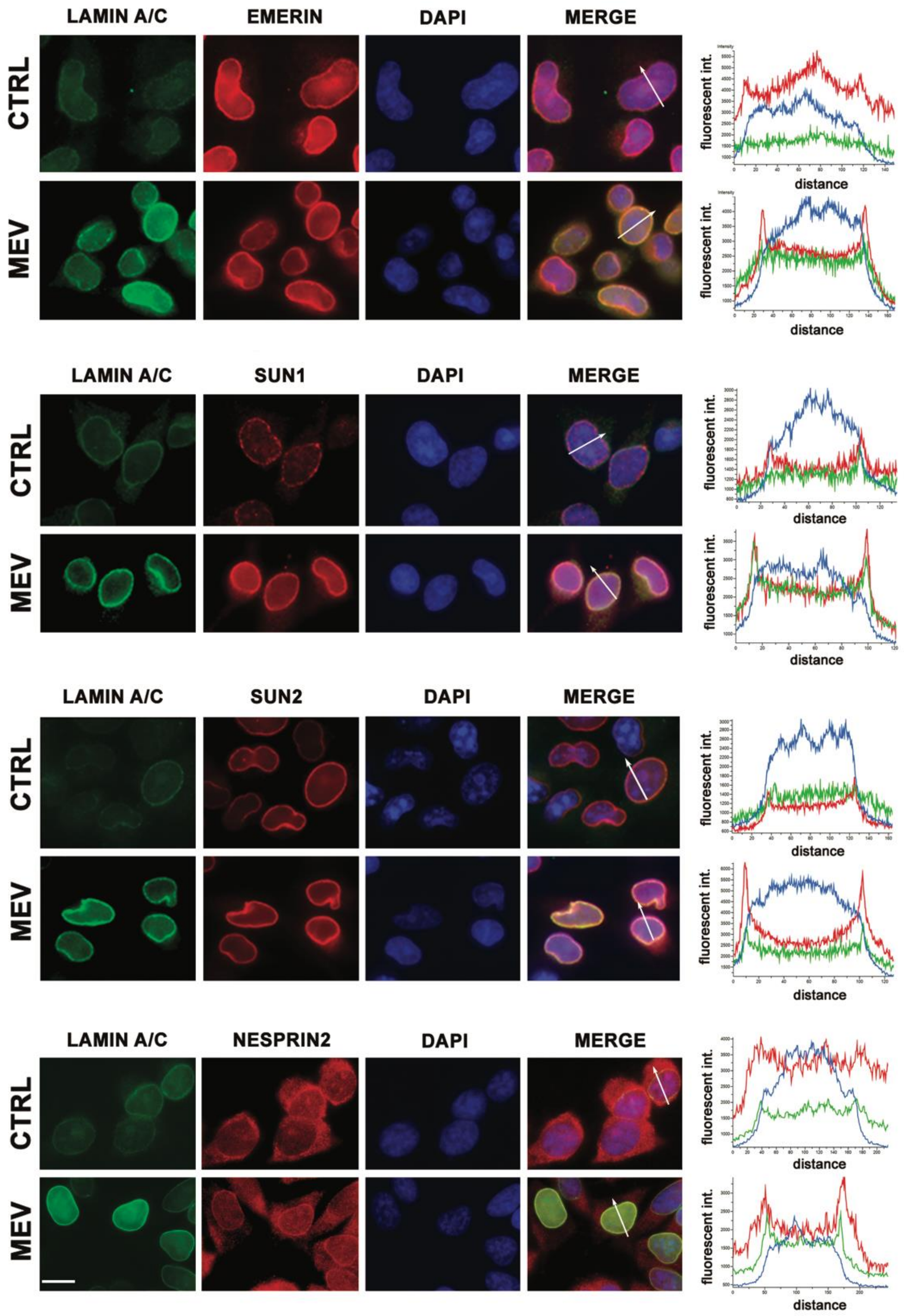

Supplementary figure 5: Lamin A/C (green), Emerin (red), SUN1 (red), SUN2 (red), and Nesprin2 (red) localization in non-treated A-673 cells (CTRL) and mevinolin treated (5 μM) EWS cells (MEV). DNA was counterstained with DAPI (DAPI). Merge of fluorescence signals are shown (MERGE). Graphs indicate the fluorescence intensity profile along the white arrows. Representative graphs of at least 30 nuclei analyzed for each sample were shown.

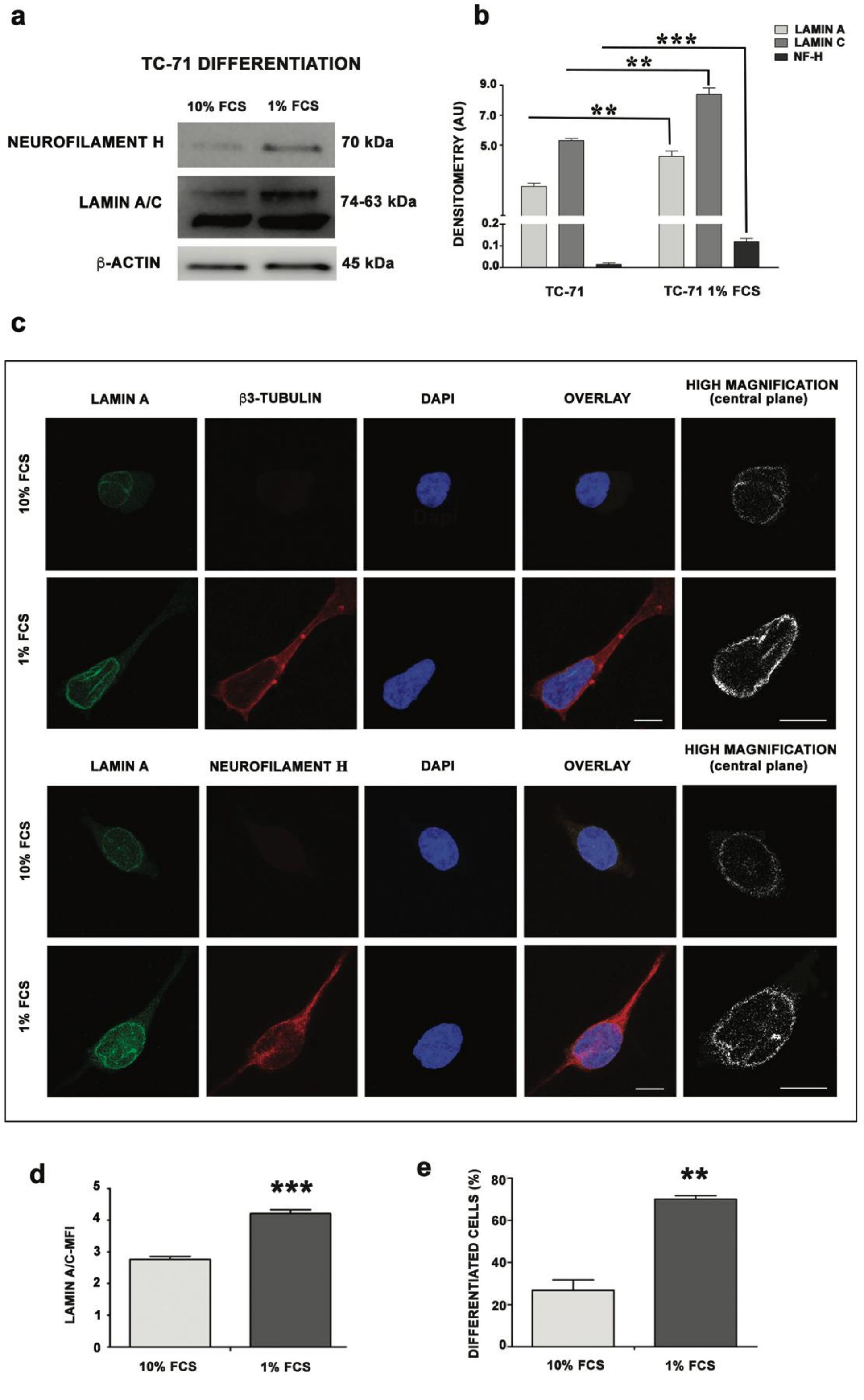

Supplementary figure 6: Low-serum conditions drive EWS cells toward neural differentiation. (**a**) Western blotting analysis of Neurofilament-H and lamin A/C protein expression in TC-71 cultured in 10% FCS medium or in 1% FCS medium. β-actin was used as loading control; (**b**) Densitometric analysis is shown as mean values ± SD of three different experiments. Asterisks indicate statistically significant differences with respect to TC-71 cultured in 10% FCS medium;

two-tailed unpaired Student’s t-test, **p<0.01, ***p<0.00; (**c**) Confocal microscopy analysis of lamin A (green), β3-tubulin (red) and Neurofilament-H (red) proteins in TC-71 cell lines cultured in 10% FCS medium or in 1% FCS medium. DNA was counterstained with DAPI (DAPI). Merge of fluorescence signals are shown (OVERLAY); (**d**) Mean fluorescence intensity (MFI) of lamin A/C immunoexpression in TC-71 cells cultured in 10% or in 1% FCS medium. Histograms were plotted as mean ± SD of three independent experiments Asterisks indicate statistically significant differences with respect to TC-71 cultured in 10% FCS medium; two-tailed unpaired Student’s t-test, ***p<0.001; (**e**) Histograms indicate the percentage of differentiated cells in 1% FCS cultured TC-71 cells. Graphs were plotted as mean ± SD of three independent experiments Asterisks indicate statistically significant differences with respect to 10% FCS cultured TC-71 cells; two-tailed unpaired Student’s t-test, **p<0.01.
