# Supplementary material for: Lamin A and the LINC complex act as potential tumor suppressors in Ewing Sarcoma
Source: Cell Death Dis. 2022 Apr 14;13(4):346. doi: 10.1038/s41419-022-04729-5 (PMC9010457; doi:10.1038/s41419-022-04729-5)

UNEDITED BLOT FOR FIGURE 1

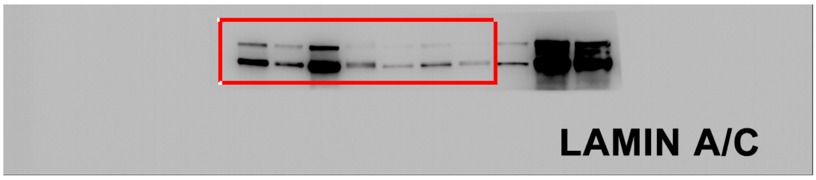

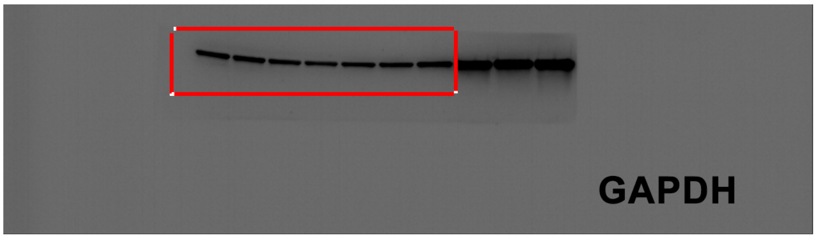

UNEDITED BLOT FOR FIGURE 4

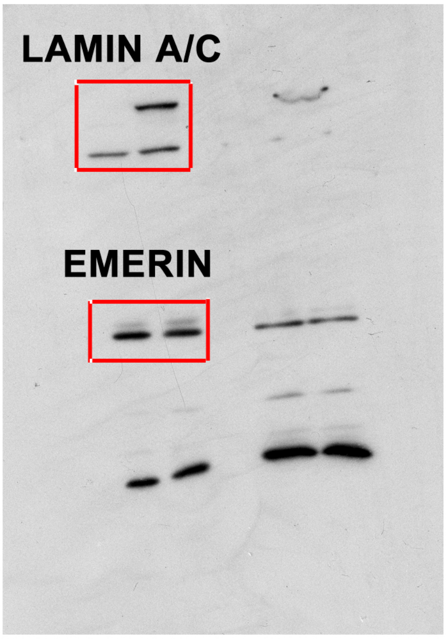

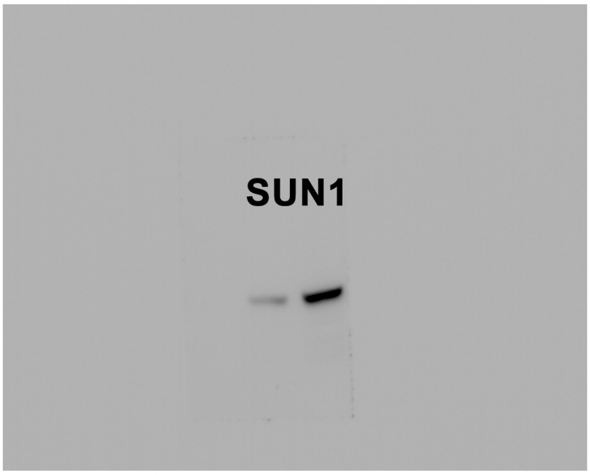

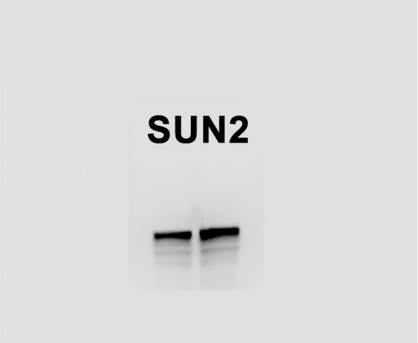

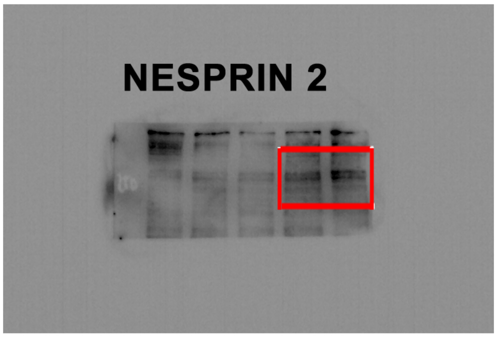

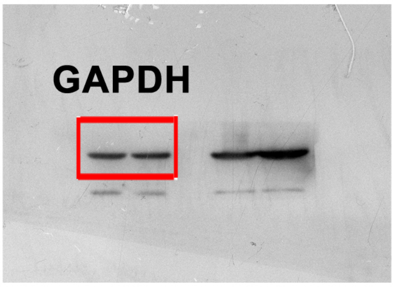

UNEDITED BLOT FOR FIGURE 5B

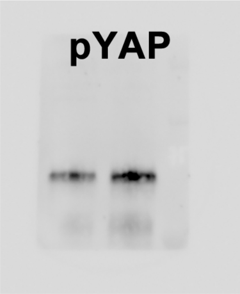

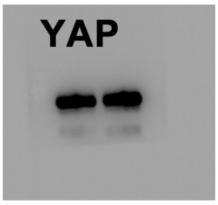

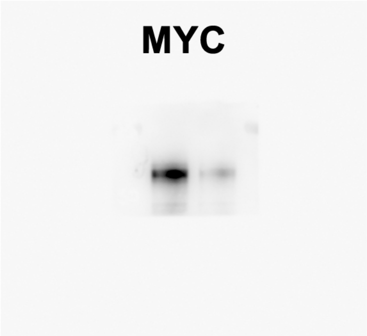

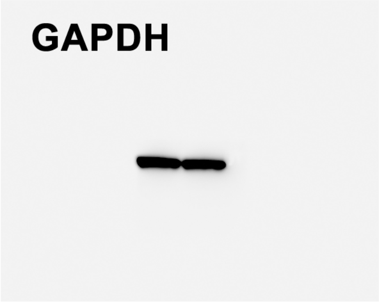

UNEDITED BLOT FOR FIGURE 5C

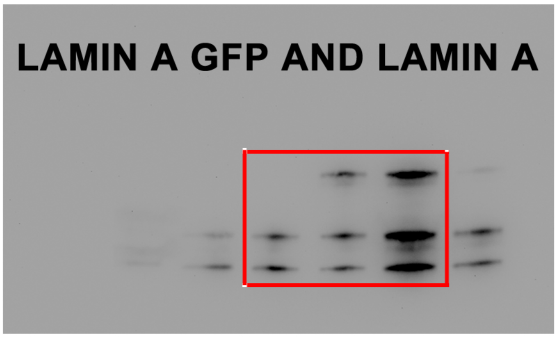

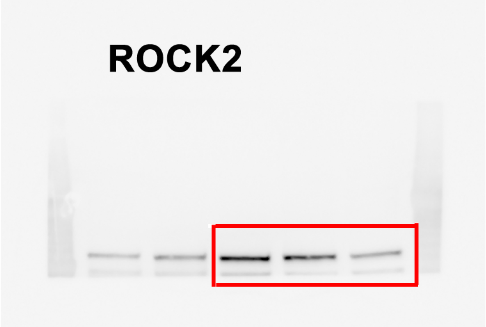

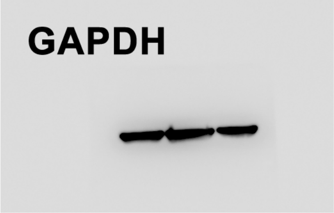

UNEDITED BLOT FOR FIGURE 5D

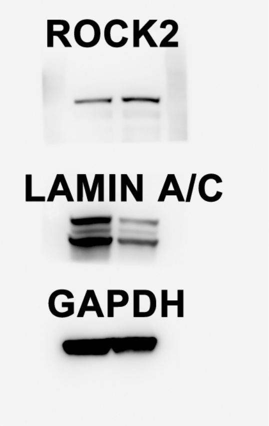

UNEDITED BLOT FOR FIGURE 6A

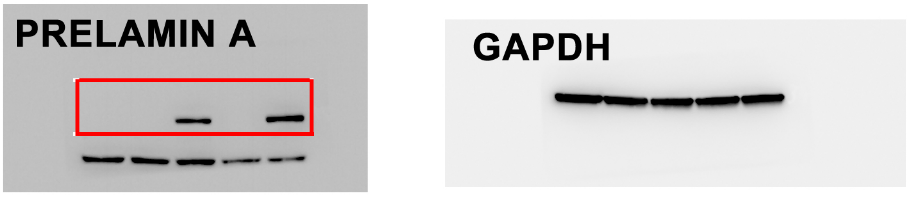

UNEDITED BLOT FOR FIGURE 6E

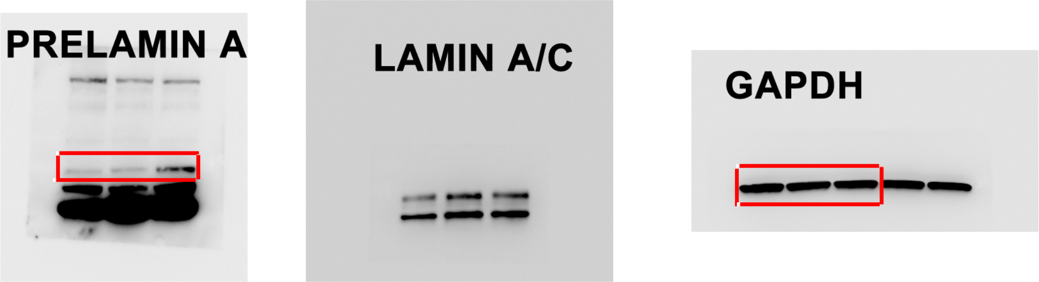

UNEDITED BLOT FOR FIGURE 6L

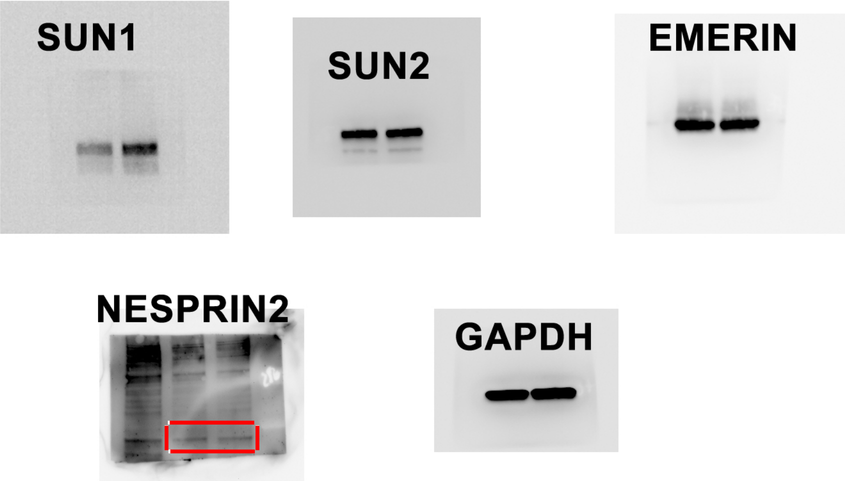

UNEDITED BLOT FOR FIGURE 7

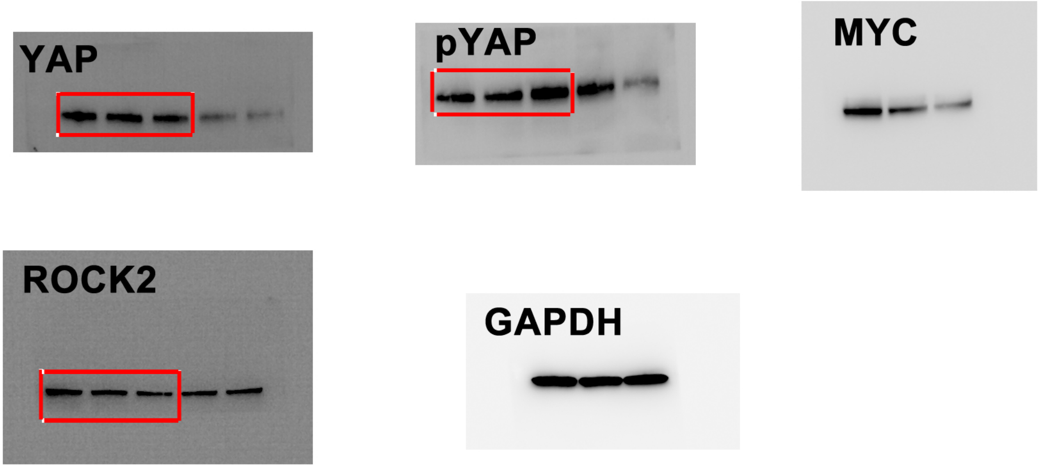

Supplement: Supplementary file 3 — Western blots all figures [file 41419_2022_4729_MOESM3_ESM.pdf]
